# Phase shift between joint rotation and actuation reflects dominant forces and predicts muscle activation patterns

**DOI:** 10.1093/pnasnexus/pgad298

**Published:** 2023-10-10

**Authors:** G P Sutton, N S Szczecinski, R D Quinn, H J Chiel

**Affiliations:** School of Life Sciences, University of Lincoln, Lincoln LN6 7TS, UK; Department of Mechanical and Aerospace Engineering, West Virginia University, Morgantown, WV 26506-6106, USA; Department of Mechanical Engineering, Case Western Reserve University, Cleveland, OH 44106, USA; Department of Biology, Case Western Reserve University, Cleveland, OH 44106, USA; Department of Neuroscience, Case Western Reserve University, Cleveland, OH 44106, USA; Department of Biomedical Engineering, Case Western Reserve University, Cleveland, OH 44106, USA

## Abstract

During behavior, the work done by actuators on the body can be resisted by the body's inertia, elastic forces, gravity, or viscosity. The dominant forces that resist actuation have major consequences on the control of that behavior. In the literature, features and actuation of locomotion, for example, have been successfully predicted by nondimensional numbers (e.g. Froude number and Reynolds number) that generally express the ratio between two of these forces (gravitational, inertial, elastic, and viscous). However, animals of different sizes or motions at different speeds may not share the same dominant forces within a behavior, making ratios of just two of these forces less useful. Thus, for a broad comparison of behavior across many orders of magnitude of limb length and cycle period, a dimensionless number that includes gravitational, inertial, elastic, and viscous forces is needed. This study proposes a nondimensional number that relates these four forces: the phase shift (*ϕ*) between the displacement of the limb and the actuator force that moves it. Using allometric scaling laws, *ϕ* for terrestrial walking is expressed as a function of the limb length and the cycle period at which the limb steps. Scale-dependent values of *ϕ* are used to explain and predict the electromyographic (EMG) patterns employed by different animals as they walk.

**Significance Statement**

There have been many discussions about how scaling in locomotion changes the relationship between inertial, gravitational, viscous, and elastic forces, with inertial forces governing motion in large limbs and elastic forces governing small limbs. We show that regimes of differing force dominance require the nervous system to solve differing control problems and show that the dominant regime of a movement can be determined by measuring a single parameter: the phase relationship between muscle force and joint angle. This work shows locomotion of large fast creatures (e.g. horses) and small slow creatures (e.g. snails) exists upon a continuum that can be evaluated and represented by this single dimensionless parameter. This will greatly inform and aid locomotion researchers in the study of locomotion.

## Introduction

Brains are embodied (1). Consequently, the mechanics of the body provide both opportunities for and constraints on the nervous system. Thus, while it is natural to consider how evolutionary pressures shape neural structures that control behavior, such pressures can only be fully understood within the context of an animal's mechanics. Muscles apply forces within the body; how do those forces interact with the mechanical properties of the limb (inertia, gravity, elasticity, and viscosity) as a function of size and speed?

To better understand how muscle force interacts with limb mechanical properties, studies often employ dimensionless numbers that express the ratio of two forces within a particular motion. For example, the Froude number, which is the ratio of centripetal inertial force to gravitational force acting on the body of a locomoting animal, has been shown to predict the gait (2, 3), duty factor, and stride length (4) of many large animals as they walk and run. As another example, the Reynolds number, which is the ratio between the inertial force and viscous force of fluid acting on an animal as it moves, predicts whether the animal should swim most efficiently through that fluid via a corkscrew or paddling motion (5). These dimensionless numbers, although extremely useful, are subject to certain assumptions and limitations that prevent their application to all systems. Specifically, each is best applied to a system whose motion is dominated by the two forces compared by the dimensionless number. For example, if viscous forces are much larger than both centripetal and gravitational forces, Froude numbers will not reflect major portions of the movement. Likewise, if gravitational forces are much larger than inertial or viscous forces, Reynolds numbers will not reflect useful insights about a movement.

Which forces dominate a given behavior, however, is governed by the scale and speed of the movement. For example, because mass scales with the cube of length, mass-dependent forces dominate in large animals (6, 7), whereas elastic forces, which scale with the square of length, dominate in small animals (8–10). Similarly, the timing of a movement also affects the forces that dominate. In an oscillatory movement like walking, because elastic forces are a function of position, they are thus insensitive to movement speed, whereas inertial forces are proportional to acceleration, and consequently the faster the oscillation, the greater the inertial forces will be relative to the elastic forces. This has major consequences for the neural control of these movements: for large animals, neural output is required to react to gravitational and inertial forces, whereas, for small animals, gravity and inertial forces can be disregarded (10, 11), with the nervous system instead needing to react to the increased role of elastic forces. Changes in the dominant force in turn change how best to quantify and control movement in animals of differing sizes and speeds, and these relationships have been quantified in some models (e.g. insect walking versus horse walking) (10–12). Similarly, internal viscous forces of a joint (damping) would also affect movement (10, 13), so that the behavior of smaller and faster animals is more dominated by damping than that of larger and slower animals.

Consequently, a broad comparison of movement across many orders of magnitude of limb length cannot be done with dimensionless numbers that specify the ratio of only two forces. Comparing movements across a wide range of sizes and speeds requires a dimensionless number that includes gravitational, inertial, elastic, and viscous forces, allowing comparison of the relative magnitudes of all these forces to determine which best quantifies a behavior. In this study, we propose such a dimensionless number that relates gravitational forces, inertial forces, elastic forces, and viscous forces: the phase shift, *ϕ*, between actuator force and limb displacement.

**Fig. 1.**
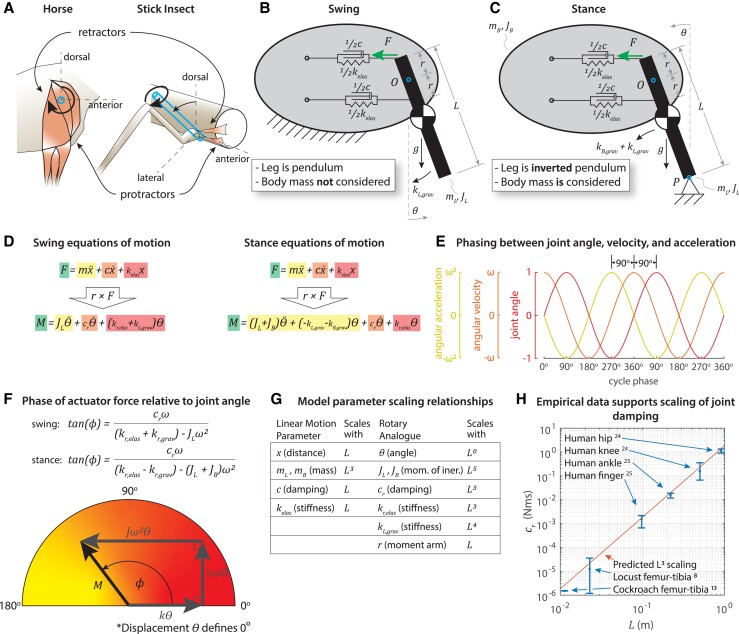
Formulation of the model. A) Horses, stick insects, and many animals possess rotary joints actuated by antagonistic muscle groups. B) In swing, we model the leg as a pendulum rotating about a fixed point O and actuated by a force F. The joint possesses intrinsic viscoelasticity, and gravity pulls the leg's center of mass (white and black circle) downward. C) In stance, we model the leg as an inverted pendulum (14–16) anchored at point P with the same mass and viscoelastic joint properties, plus the mass of the body concentrated at point O. Gravity pulls the leg's center of mass and the body's center of mass downward. D) The equations of motion in swing and stance can be written in terms of x, the actuator position. The rotary pendular dynamics is computed from the torque, i.e. the cross product of *r* with the equations of motion. Although rotating the joint will change the moment arm vector, this effect is negligible for joint motions less than 30° in either direction (Fig. S2). Each term is color coded: inertial forces and moments are yellow (in greyscale: light grey), viscous forces and moments are orange (in greyscale: medium grey), and elastic forces and moments are red (in greyscale: dark grey). Gravitational moment is shaded red in swing and yellow in stance to indicate that it acts in phase with motion (like elastic force) in swing and out of phase with motion (like inertial force) in stance. This color scheme will be used throughout the manuscript. E) When the joint angle θ(t)=sin(ωt), the angular velocity leads the displacement by 90° of the cycle period and the angular acceleration leads the displacement by 180° (i.e. out of phase). F) The phase shift *ϕ* between the actuator moment *M* and the joint angle *θ* can be represented graphically. G) Allometric scaling laws enable phase shift *ϕ* to be expressed in terms of the limb length *L* and the cycle period *T*. H) Our allometric scaling law for joint damping is consistent with literature references cited in the figure (see also Table S1). See Tables S1 and S3 for parameters and Figs. S1 to S6 for more details and parameter sensitivity analysis.

We quantify how forces are partitioned among gravitational, elastic, viscous, and inertial forces during simulations of legged locomotion (including stance and swing), finding that the relationships between these forces can be reflected by a single measurable nondimensional number: the phase shift (*ϕ*) between actuator force and limb displacement (Fig. 1). Using allometric scaling laws, we expressed *ϕ* in terms of two quantities: limb length and cycle period. As a result, we identified three “regions” of limb length and cycle period in which actuator force is primarily resisted by gravitational forces, inertial forces, elastic forces, or viscous forces. We will use swing and stance of simulated locomotion to show that, while the dominant force in a given motion is dependent on the size and frequency of the movement, the relationship between phase shift (ϕ) and the dominant force remains the same in both swing and stance, demonstrating that phase shift can be used to quantify the dominant force in a behavior.

Moreover, phase shift (ϕ) can also predict two more aspects of the movement. First, the phase shift can predict the limb's response to perturbation; and second, the phase shift can predict the timing of electromyography (EMG) during a limb movement. Since the nervous system must activate muscles with an appropriate timing relative to limb position to generate a movement, the EMG must also be shifted by *ϕ*. We test this prediction by demonstrating how the phase shift (ϕ) can predict the EMG recordings of locomotion (swing and stance) at two different speeds in two very differently sized animals: horse and stick insect.

## Results

To develop a dimensionless number for limb movement, we created a model that represents the simplified geometry and dynamics of a limb segment in both swing and stance, e.g. a horse's foreleg rotating about the shoulder or an insect's leg rotating about its thoraco-coxal joint (Fig. 1A). We model the leg of a walking animal as a rigid pendulum in swing and an inverted pendulum in stance (14–17). The limb is moved by an antagonistic pair of actuators, e.g. a shoulder protractor (flexor) and retractor (extensor). Together, the actuators have total inherent elastic stiffness *k*_elas_ and viscous damping *c*, resulting in elastic and viscous moments about the shoulder. The model is concerned with which forces resist actuator work so the actuators themselves do not include muscle dynamics, e.g. force–velocity limits (see supplementary materials for further justification of this simplification), although muscle properties limit what motions an animal can execute volitionally (18). The dynamics of the leg–body system depend on whether the leg is in swing, during which the leg is moved anteriorly (i.e. protracted) and does not support the body (Fig. 1B), or stance, during which the leg is moved posteriorly (i.e. retracted) while supporting and propelling the body (Fig. 1C). In both cases, the limb is assumed to have length *L* with mass $m_{L}$ and moment of inertia about the hip $J_{L}$. The limb is assumed to operate in a gravitational field with acceleration *g*, and the rotation of the limb relative to the direction of gravity is measured by *θ*. As in Hooper and Alexander (11, 15), we neglected aerodynamic drag.

To calculate the ratios of inertial, elastic, gravitational, and viscous forces within the limb, we applied allometric scaling relationships to express the inertia, gravitational forces, elastic forces, and viscous damping in terms of limb length (Fig. 1G) and limb movement speed. For example, a limb's mass very nearly scales with its volume, that is, its length cubed (19, 20). Similar scaling laws describe how spring stiffness scales proportional to length (21, 22). Because we were unaware of an established allometric scaling relationship for joint damping as a function of leg length, we developed one using previously published data from studies in human, stick insect, and cockroach joints (8, 13, 23–25). We found that joint damping, like spring stiffness, scales proportional to length and predicts the values for joint damping reported for limb lengths spanning two orders of magnitude (Fig. 1H). These relationships were extended to account for the rotational motion of a joint by the principle of virtual work (26) (see the supplementary materials).

**Fig. 2.**
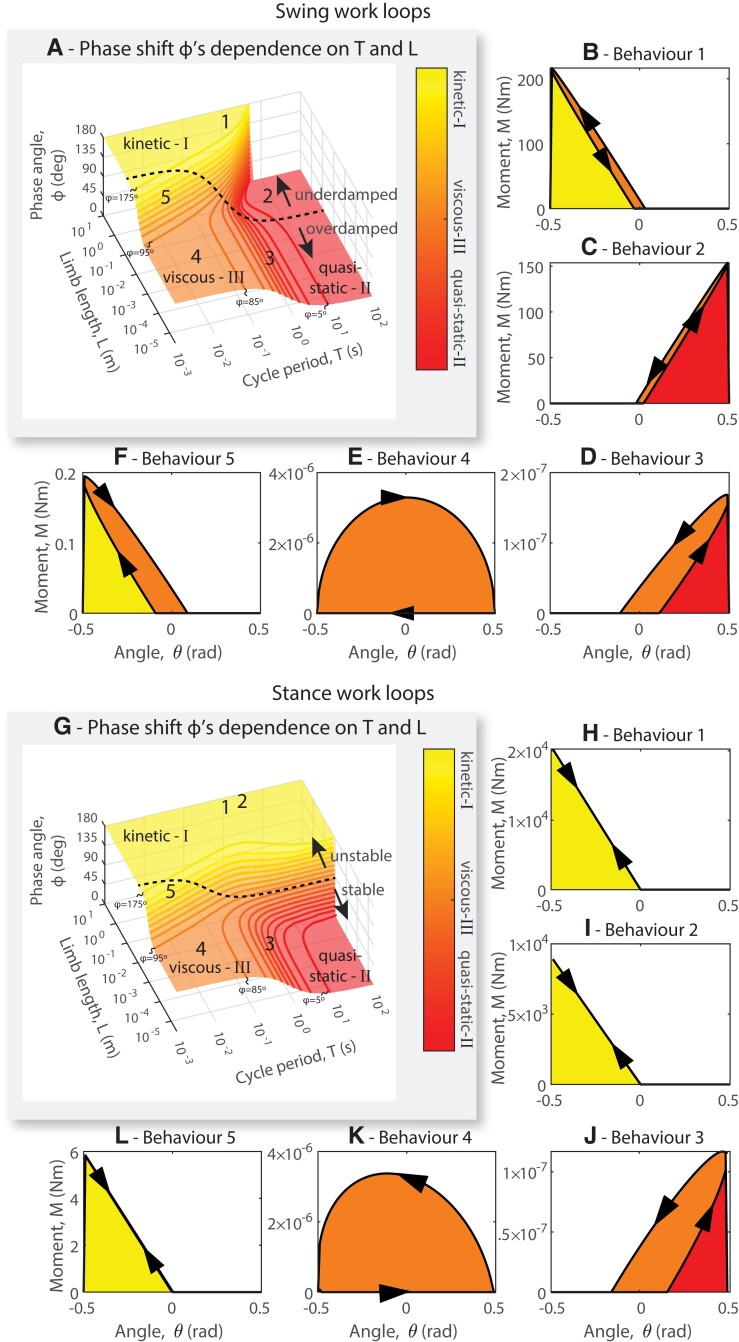
Muscle forces are resisted by inertial (kinetic zone), elastic (quasi-static), or viscous forces depending on limb length and cycle period of stepping. A) Swing phase: Each contour of constant phase shift *ϕ* (in 10° increments) represents identical distributions of inertial, elastic, and viscous forces during *swing*. Three regions (I, II, and III) appear in this plot corresponding to the plateaus in the figure. B) Work loop plotting the moment applied to the joint by one antagonistic actuator versus the joint angle. Areas below the curve represent energy added to the body. Areas within the loop represent energy dissipated due to viscosity. In each plot, the joint sweeps a range of 1 radian (approximately 60°) symmetrically about 0. In behavior 1, most actuator work is converted into kinetic energy, indicated by the large yellow region. C) In behavior 2, most actuator work is stored as potential energy, indicated by the large red region. D) In behavior 3, most actuator work is stored as potential energy and a noticeable amount is dissipated by viscosity, indicated by the orange region. E) In behavior 4, most actuator work is dissipated by viscosity. F) In behavior 5, most actuator work is converted into kinetic energy. G) Stance phase: Each contour of constant phase shift *ϕ* (in 10° increments) represents identical distributions of muscle work into kinetic, viscous, and potential energy during *stance*. H) In behavior 1, most actuator work is converted into kinetic energy, similar to what is observed in swing. I) In behavior 2, most actuator work is converted to kinetic energy, in contrast to the energy distribution in swing (C). J) In behavior 3, most actuator work is converted to potential energy. K) In behavior 4, most actuator work is dissipated due to viscosity. L) In behavior 5, most actuator work is converted into kinetic energy.

In swing, the phase shift (*ϕ*) between force and movement is determined by limb length and cycle period. In turn, the phase shift quantifies the ratio of the inertial, gravitational, potential, and viscous forces, as shown in Fig. 2A. Although the phase shift varies continuously with limb length and cycle time, there are regions in which large changes in the phase shift occur over small changes in cycle time or length, and these determine distinct “regions.” In region I (yellow), the cycle period is so short relative to the limb's natural period of oscillation that the actuator force is almost entirely out of phase with the motion, resulting in a phase shift (*ϕ*) of 180°. This is indicated by the work loops (27) shown in Fig. 2B and F, which plot the actuator force versus the limb angle. We call this region “kinetic” because most of the actuator work is resisted by inertial forces and is thus converted into kinetic energy (represented by the yellow shaded area). In region II (red), the cycle period is longer than the limb's natural period, and actuator torque is almost entirely in phase with the limb angle, as indicated by the positive-slope work loops shown in Fig. 2C and D. The phase shift (*ϕ*) in this region is 0°. We call this region “quasi-static” because the static forces of gravity and elasticity dominate (28), and most actuator work is converted into potential energy (shaded red). Finally, in region III (orange), cycle period is short relative to the resonant frequency of the limb, but the limb has very little mass, so most actuator energy is dissipated due to viscous forces within the joint (orange shading in Fig. 2E). The phase shift in this region is intermediate but usually near 90°. For swing at all sizes and cycle times, the phase shift (*ϕ*) indicates whether the motion is dominated by inertia, gravity and elasticity, or viscosity.

In stance, the relationship between limb length, cycle period, and phase shift changes dramatically compared to swing, as indicated by Fig. 2G. The primary difference is that movements that previously resided in region II now reside in region I (the kinetic region), meaning that the phase shift changes substantially between swing and stance in some cases. Specifically, for relatively slow motions in relatively large animals, swing would be quasi-static but stance would be kinetic. This change occurs because gravity dominates at this limb length and cycle period, and although it stabilizes the leg in swing, it destabilizes the body's posture during stance, changing the phase shift of the gravity term by 180°. This transition is evident when comparing Fig. 2C and I. In our simulated behaviors, slow movements for large animals have a phase shift of 0° in swing (Fig. 2A, behavior 2) and a phase shift of 180° in stance (Fig. 2G, behavior 2). For both stance and swing, however, the relationship between phase shift and the dominant forces within the movement remains the same. There are still three regions (inertia and gravity dominated, quasi-static, and viscous dominated), with a 180° phase shift indicating inertially or gravitationally dominated movements, a 0° phase shift indicating elastically dominated movements and a 90° phase shift indicating viscously dominated movements.

**Fig. 3.**
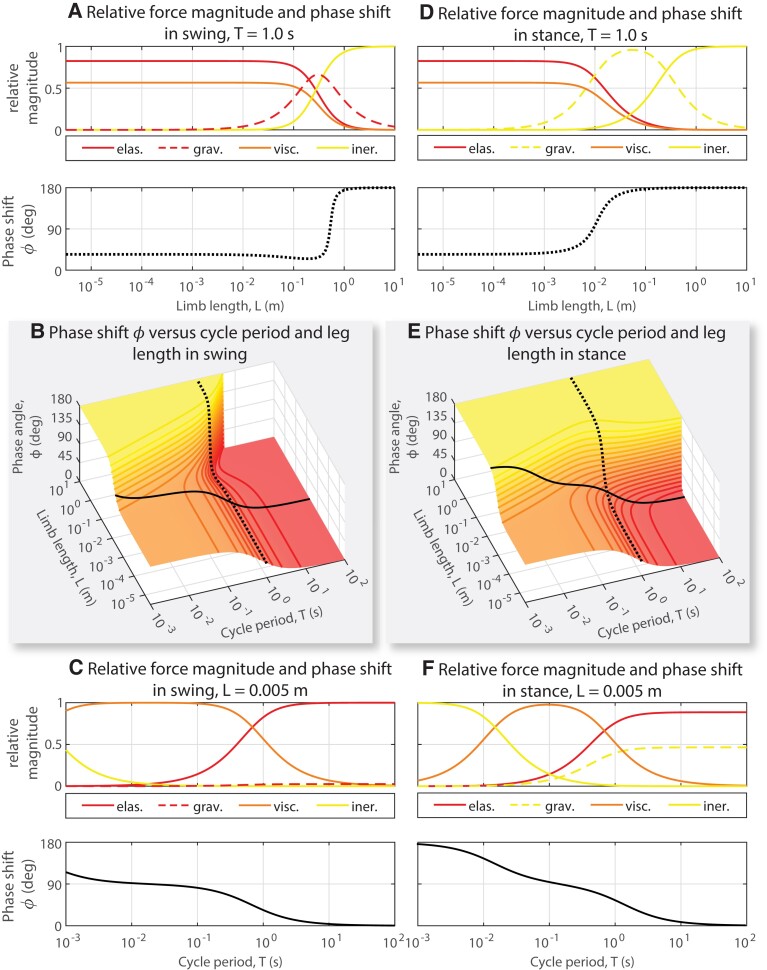
The relationship between phase shift and dominant force can be illustrated by looking at cut throughs of the phase shift as either time or length is held constant and the other is varied. A) At a constant cycle period of 1 s under swing phase conditions, the motion of small limbs (*L* < 10^−1^ m) is dominated by elastic and viscous forces, but the motion of large limbs (*L* > 1 m) is dominated by inertial force. This is reflected by the corresponding plot of phase shift *ϕ*, which is near 45° for small limbs and near 180° for large limbs at this cycle period. B) Plot of the full *ϕ* versus *T* and *L* landscape for swing. The color shading and contours are the same as in Fig. 2A. Cut throughs are indicated by dotted (constant period *T* = 1 s) or solid (constant length *L* = 0.005 m) black lines. C) For a limb of length 0.005 m, rapid oscillations (*T* < 10^−1^ s) are dominated by viscous force and slow oscillations (*T* > 10 s) are dominated by elastic force. This is reflected by the corresponding plot of phase shift *ϕ*, which is near 90° for rapid oscillations and near 0° for slow oscillations. D) Same analysis as in A, but for stance phase conditions, mass is much greater and gravity is shifted 180° relative to the joint angle (indicated by change from red to yellow dashed line). Because of gravity's phase shift, *ϕ* approaches 180° at shorter limb lengths than in swing. E) Plot of the full *ϕ* versus *T* and *L* landscape for stance. The color shading and contours are the same as in Fig. 2G. Cut throughs are indicated by dotted (constant period *T* = 1 s) or solid (constant length *L* = 0.005 m) black lines. F) Same analysis as in C, but for stance phase conditions. Inertial force dominates extremely rapid oscillations (*T* < 10^−2^ s), viscous force dominates intermediate-speed oscillation (10^−2^ s < *T* < 1 s), and elastic force dominates slow oscillations (*T* > 1 s). Note that at slow oscillations, the contribution of stabilizing elastic forces is greater than that from destabilizing gravitational forces.

The relationship between phase shift and dominant force can be illustrated more thoroughly by looking at a cut through of the phase shift plot across time for both swing and stance. For a cycle time of 1 s, the swing phase of limbs shorter than 10^−2^ m is dominated by the elastic and viscous forces within the limb, while the swing phase of limbs longer than 10^−2^ m is dominated by the gravitational and inertial forces within the limb (Fig. 3A). Likewise, for limbs shorter than 10^−2^ m, the phase shift between actuator force and limb position is close to 0°, while for limbs longer than 10^−2^ m, the phase shift is 180°. For stance, at the same cycle time, the shift from elastically dominated forces to inertially dominated forces occurs at a length of 2 × 10^−1^ m (Fig. 3D), with the change in phase shift occurring at that length as well.

The same relationship between phase shift and dominant force in the limb is also seen when looking at a cut across length for both simulations. In swing, for a limb length of 5 × 10^−3^ m, a movement with a cycle time of 10^−2^ s is dominated by viscous forces within the limb, with a transition to elastic force dominance when the cycle time becomes longer than 1 s (Fig. 3C). As the limb movement is more and more dominated by elastic forces, the phase shift changes from near 90° to near 0°. In stance, at the same length scale (Fig. 3F), the relationship between cycle time and the dominant limb force is very different than for swing, with inertial forces being dominant at cycle times of less than 10^−2^ s and elastic forces becoming dominant at cycle times greater than 10^0.5^ s (Fig. 3F). Despite the difference in which forces are present, the relationship between phase shift and the dominant force is the same in stance as for swing, with a phase shift of 180° reflecting the dominance of inertial forces, a phase shift of 0° reflecting the dominance of elastic forces, and a phase shift of 90° reflecting the dominance of viscous forces. The phase shift between actuator force and limb angle thus illustrates which forces are dominant within the limb, for both stance and swing, at a wide range of length and time scales. For all simulations, a phase shift of 180° indicates dominant inertial forces (the kinetic region), a phase shift of 0° indicates dominant elastic forces (the quasi-static region), and a phase shift of 90° indicates dominant viscous forces. The only difference between these movements (i.e. stance and swing) is the direction of gravity; if gravity stabilizes the motion, its phase shift is 0°; if gravity destabilizes the motion, its phase shift is 180°. If the phase shift can be precisely measured, intermediate values of phase shift can also indicate relative magnitudes of these forces: for example, in stance at a length of 5 × 10^−3^ m (Fig. 3F), at a cycle period of 7 × 10^−1^ s, the viscous forces and elastic forces are equal in magnitude, with a commensurate phase shift of 45°, exactly halfway between a phase of 0° (elastic force dominance) and 90° (viscous force dominance).

**Fig. 4.**
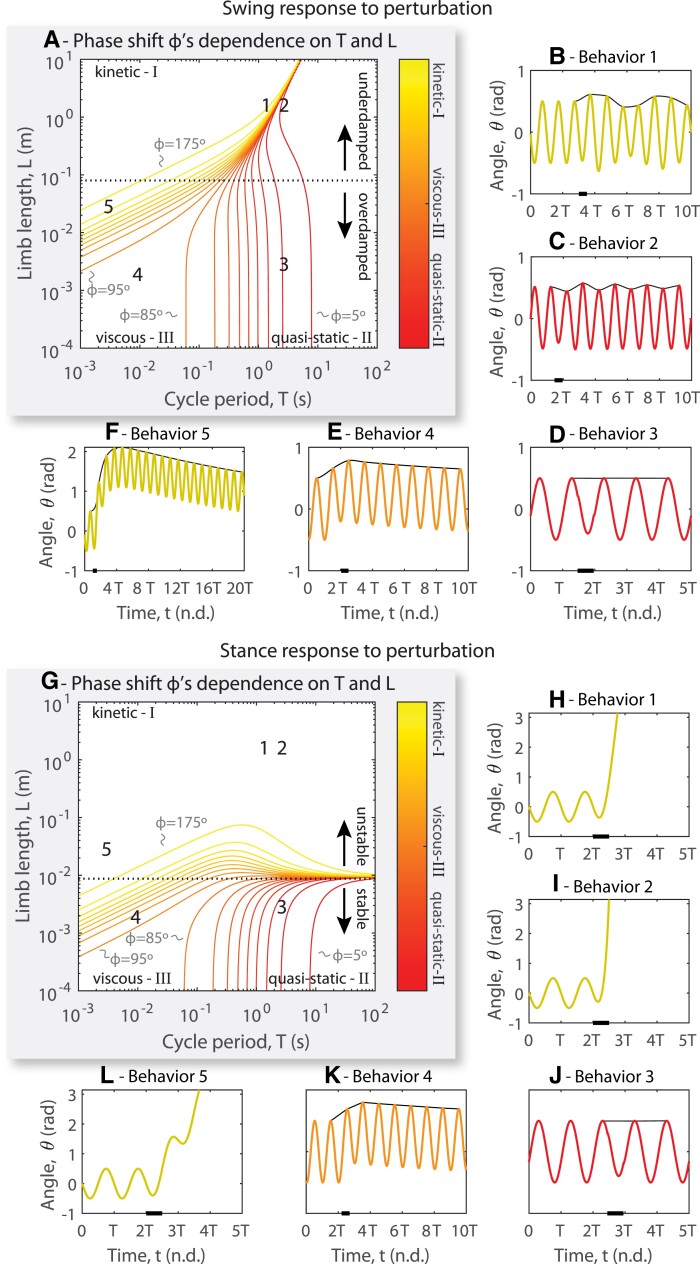
Muscle forces are resisted by differing amounts of inertial, viscous, elastic, and gravitational forces depending on limb length and cycle period of stepping. A) Swing phase: The plot from Figs. 2A and 3B, “flattened” into two dimensions (see also Fig. S5). Each contour of constant phase shift *ϕ* (in 10° increments) represents identical distributions of muscle work into kinetic, viscous, and potential energy during *swing*. Three regions appear in this plot corresponding to the regions in Fig. 2. B) To test the response to perturbation, we applied a perturbation equal to 20% of the magnitude of the steady-state actuation torque for one half of a cycle period (black bar on time axis). We then plot the subsequent joint angle versus time. To track how the amplitude varies from cycle to cycle, the maximum angle reached during each cycle is traced with a spline (solid black line in each plot). In behavior 1, a small perturbation alters the motion in a highly erratic way for many subsequent cycles because the energy cannot be easily dissipated. C) In behavior 2, energy is dissipated rapidly compared to the natural period of oscillation and the system returns to its previous oscillatory pattern. D) In behavior 3, the system does not oscillate because it is overdamped. E) In behavior 4, a perturbation does not cause erratic oscillation as in B, but it does alter the mean angle of the ongoing motion. F) In behavior 5, a perturbation substantially alters the mean angle of the ongoing motion but does not cause erratic oscillation as in B because it is overdamped. G) Stance phase: The plot from Figs. 2G and 3E, “flattened” into two dimensions (see also Fig. S5). Each contour of constant phase shift *ϕ* (in 10° increments) represents identical distributions of muscle work into kinetic, viscous, and potential energy during *stance*. In behaviors 1 (H), 2 (I), and 5 (L), unlike in swing, static posture is unstable. J) In regions 3 (J) and 4 (K), static posture is predicted to be stable because elastic torques at the hip are greater than those from gravity.

Recognizing the phase shift and the scale dependence of inertia, gravity, elasticity, and damping also has major consequences for how a simulated limb movement reacts to a perturbation. Regions I and II can be divided between two areas, one in which the limb is mechanically underdamped (examples seen in behavior 1 [kinetic underdamped] and 2 [quasi-static underdamped]), and one in which the limb is mechanically overdamped (example seen in behavior 5 [kinetic overdamped] and behavior 3 [quasi-static overdamped]). Depending on the region of a given movement, responses may be stable overdamped (a perturbation is quickly removed from the system through damping), stable underdamped (a perturbation is eventually removed from the system, but there are multiple oscillations), or unstable (perturbations lead to uncontrolled movements). In our swing simulations, all responses are stable, with large limbs being underdamped and small limbs being overdamped. For large limbs in region I, perturbation causes lasting alterations to the ongoing motion unless excess kinetic energy is absorbed by the actuator (Fig. 4B and C). However, for small limbs, the damping parameter (Fig. 1H) is large enough that the joint will rapidly dissipate excess kinetic energy (Fig. 4E and F). Large amounts of kinetic energy can only be built up, however, when inertial forces are dominant (i.e. when the phase shift is 180°). When the phase shift is 90° or less, such as for motions with long cycle periods (i.e. quasi-static motions), energy is dissipated quickly relative to the cycle period, implying that perturbations may not have a noticeable effect on the limb's motion (Fig. 4D).

In stance, the kinetic region, where the phase shift is 180°, is quite large, and in most of this area, the posture is unstable. The model predicts that locomotion with limbs longer than about 1 cm is unstable, implying that the locomotion of animals larger than 1 cm will destabilize in response to perturbation if no feedback control is used. This results in the limb angle “exploding” (i.e. the animal falls down) if it is perturbed (Fig. 4H, I, and L). Interestingly, the posture of animals with limbs shorter than about 1 cm is expected to be passively stable, implying that an animal could stand up without any active muscle contraction as long as their feet do not slip on the substrate. Animals with short limbs could also passively reject perturbations during stance (Fig. 4J and K).

**Fig. 5.**
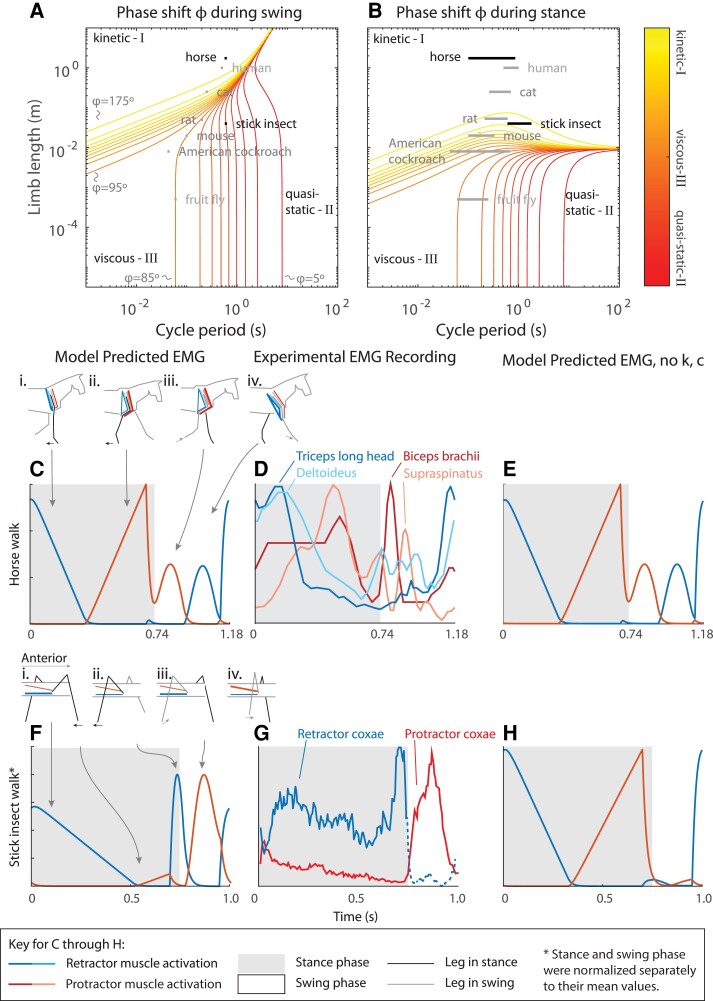
Our model predicts the disparate EMG patterns observed in animals of very different sizes. A) The phase shift during *swing* is plotted (0° in red, 90° in orange, and 180° in yellow), along with the duration of swing and leg length of several species (horse (29), human (30), cat (31), rat (32), stick insect (33), mouse (34), American cockroach (35), and fruit fly (36)) Numerical values are presented in Table S2. Animals should require region-specific motor patterns to accomplish the same motion. B) The phase shift during *stance* is plotted, along with the reported range of stance duration and leg length of the same species as in A. This shows that for the same length and cycle period, stance and swing can have different phase shifts. C) For horse, the model predicts decreasing retractor activity during the first half of stance (inset: stance leg black, swing leg gray, retractor muscle blue, and protractor muscle red; gray background stance and white background swing), bimodal protractor activity straddling the stance-swing transition, and reactivation of retractor muscles at the end of swing. D) Averaged EMG recordings from three thoroughbred horses walking with mean stance phase duration 0.74 s and mean swing phase duration 0.44 s (29). Harrison et al. (29) classify the *triceps brachii's* long head and *deltoideus* muscles as shoulder flexors that retract the foot and the *biceps brachii* and *supraspinatus* as shoulder extensors that protract the foot. The scale of each muscle's EMG was normalized to the maximum reading during a canter gait by Harrison et al. (29). E) The model's prediction does not visibly differ from a model in which no elastic or viscous forces are present, which is consistent with a horse's large size. F) For an animal on the scale of a stick insect, the model predicts decreasing retractor muscle activity throughout stance, a brief burst of retractor muscle activity at the end of stance, and almost exclusively protractor muscle activity during swing (inset: stance leg black, swing leg gray, retractor muscle blue, protractor muscle red, gray background stance, and white background swing). G) Averaged EMG recordings from 174 steps from five stick insects walking unsupported at their preferred speed (steps at 1 Hz, 70% in stance phase) (37). The coxal retractor (blue) and coxal protractor (red) actuate the shoulder-like thorax-coxa joint during walking. Note that this EMG pattern differs substantially from the horse recording in Fig. 4D. The increase of EMG activity in the retractor coxae muscle (blue) observed at the end of stance (gray background) is successfully predicted by our model and is a consequence of the phase shift of stick insect locomotion representing a dominance of elastic forces. H) When the size-dependent effects of elastic and viscous forces are not considered, the predicted EMG patterns are dramatically different from the recordings, emphasizing the predictive capability of our modeling framework. Our modeling framework explains interspecies differences in the EMG patterns of walking animals and makes testable predictions for future experiments. This is, of course, with the caveat that EMG patterns can be suboptimal reflections of the forces within the system (38, 39). For more details, example joint torques for horse and stick insect are presented in Fig. S7.

Phase shift can be used to predict EMG patterns of locomoting animals. Figure 5A and B overlay the reported swing and stance durations of multiple animals’ walking on the plot of the phase shift (horse (29), human (30), cat (31), rat (32), stick insect (33), mouse (34), American cockroach (35), and fruit fly (36)). Due to the unique energetics and stability of motion within each region, all these walking motions should arise from widely varying motor output. To better understand how motor output should vary, the torques required to actuate the hip of two model organisms, horse (29) and the stick insect (37), were calculated via inverse dynamics. The calculation was performed twice: once using the full set of parameter values $J,\;c_{r},\;k_{r,\mathrm{elas}},\;$ and $k_{r,\mathrm{grav}}$ and again with $c_{r}=0$ and $k_{r,\mathrm{elas}}=0$ to determine roles of damping and elastic forces within this behavior.

EMG patterns were approximated by assuming joint torque in the retractor direction was applied by the retractor muscle and torque in the protractor direction was applied by the protractor. EMG patterns were advanced relative to the calculated torque to mimic the approximately 50-ms lag between EMG activity and muscle force production (38) (see also the supplementary materials and Fig. S7). Our approximated EMG patterns cannot account for cocontraction of antagonist muscles and primarily reflect changes in muscle activity throughout the stepping cycle.

The model predicts unique EMG recordings of hip muscle activity driving the same joint motion in two animals, the horse and stick insect. In the horse (which exists in the kinetic region, phase shift 180°), gravity dominates the stance phase, so the moment due to gravity about the foot should be counteracted by the hip retractor muscles at the beginning of stance (Fig. 5Ci) and the hip protractor muscles at the end of stance (Fig. 5Cii). The predicted activations are observed in EMG recordings from walking horses (Harrison et al. (29) and Fig. 5D). During swing, the protractor muscles initially accelerate the leg with an impulse (Fig. 5Ciii), and then the retractor muscles decelerate the leg with an opposing impulse (Fig. 5Civ). As in stance, the model prediction and experimental data confirm these activation patterns. Because horses are large, removing elastic and viscous parameters does not noticeably affect the predicted EMG (Fig. 5E).

We also used the model to predict EMG of stick insect walking, which, in contrast to the horse, exists in the quasi-static region (phase shift 0°). Due to the small size and slow movement of the stick insect, the model's prediction of its EMG pattern is dramatically different from that for horse. Figure 5B predicts stance to be nearly kinetic (inertia dominated) for the stick insect, meaning that as in the horse, stance begins with hip retractor (i.e. the thoraco-coxal retractor) activation (Fig. 5Fi). However, due to its small size, the stick insect experiences relatively large viscous moments at the shoulder, which modify the relative phase of muscle activation. Furthermore, it experiences relatively large elastic forces at the shoulder, which act opposite to gravity. Thus, near the end of the stance phase, the gravitational forces are counteracted almost entirely by elastic forces, resulting in nearly no muscle activation of the coxal retractor or protractor (Fig. 5Fii). As the swing phase begins, the foot is lifted from the substrate, and the gravitational force that had counteracted elastic force disappears, requiring the retractor to greatly increase its activation to prevent the leg from “snapping” forward like a mousetrap (Fig. 5Fiii). This unexpected feature is also observed in kinematics and experimental EMG recordings from freely walking stick insects (37) (Fig. 5G) and is a consequence of the dominance of elastic forces in stick insect locomotion. As the swing phase continues, the protractor (i.e. the thoraco-coxal protractor) activates to overcome the viscous forces that resist the swing phase motion (Fig. 5Fiv), as predicted by the phase shift during swing (Fig. 5A). Since elastic and viscous forces dominate the locomotion of small animals, removing the elastic and viscous elements from the model greatly reduces the model's prediction accuracy (Fig. 5H).

In both models of horse and stick insect locomotion, the phase shift (*ϕ*) between force and limb angle indicates which forces are most dominant during the movement and helps to explain experimental EMG patterns seen in the literature.

## Discussion

To quantify the dominant force (gravitational, elastic, viscous, and/or inertial) during a behavior, we have described a nondimensional number: the phase shift (*ϕ*) between actuator force and limb displacement (Fig. 1). Using allometric scaling laws, we expressed the phase shift (*ϕ*) in terms of two quantities: limb length and cycle period. By modeling both swing and stance for a wide range of differing limb sizes and limb cycle times, we identified “regions” of limb length and cycle period in which actuator force is primarily resisted by the body's inertia, gravitational force, elastic force, or viscous force (Fig. 2). In each region, movement has very different responses to perturbation (Fig. 4) and is driven by dramatically different patterns of force over time. However, for both swing and stance, the relationship between phase shift (*ϕ*) and the dominant force within the behavior was the same, with inertially dominated behaviors having a phase shift of 180°, quasi-static (elastically dominated) behaviors having a phase shift of 0°, and viscous dominated behaviors having a phase shift of 90° (Fig. 4). We then showed how this phase shift can be used to predict the differing EMG patterns observed for a large locomoting animal (horse) and a small locomoting animal (stick insect, Fig. 5).

Despite the value of the phase shift for understanding broad trends in the control of movement, this framework has limitations that can be addressed in future work. Because this study was based on allometric scaling of mechanical properties like mass, viscous damping, and joint stiffness, it cannot account for species-specific variations. This framework is not meant to be a replacement for species-specific investigations of the mechanical properties of animal legs (9, 10, 13, 24, 25, 40, 41). Instead, it is intended to facilitate a comparison of dynamics in legged locomotion across many different scales. Furthermore, while this framework should be broadly applicable to other periodic motions, e.g. insect flapping-wing flight (42, 43) or soft-bodied feeding (44), the parameters within this model were tuned with leg joints in mind and the model may not accurately describe these motions without some retuning of parameters. Finally, this framework treats the leg as a single rigid link (a commonly used simplification (45, 46)), despite legs utilizing many joints with coupled dynamics (47, 48). We anticipate that the basic framework presented here will lead to future studies that refine its predictions and extend its applicability to more systems.

The relationship between phase shift and dominant force results from physics and is explained by classical mechanics (Fig. 1 and Supplementary material). For any linear second-order system, these relationships will hold. Even if the values of the damping, elasticity, and inertia coefficients differ (i.e. allometric scaling laws must be adjusted), the relationship between phase shift (*ϕ*) and the dominant force within a behavior will still hold. Thus, we believe this framework could readily be extended to encompass different environmental media within which behavior occurs. An example of such a scenario would be legged locomotion through water. The water would increase the inertia and damping of the leg, but that increase in inertia and damping would in equal measure increase the phase shift between the actuator force and limb position. Similarly, at the speed at which flies flap their wings, the nonstationary dynamics of air become important (49). Once again, the framework could readily be adjusted to incorporate these environmental features, by incorporating the fluid forces that the wings must overcome, as a function of the kinematics. We believe the phase shift analysis we present could be applied to different neuromechanical systems and environments as long as the mass, stiffness, and damping parameters are adjusted to reflect that neuromechanical system and environment.

Analysis of phase shift links multiple nondimensional numbers that describe locomotion at particular scales. The interfaces between our named regions of force dominance (i.e. kinetic, viscous, and quasi-static) are not only level curves of *ϕ*; they are also level curves of other nondimensional numbers. For example, the Froude number is the ratio between inertial centripetal force and gravitational force, $Fr=\frac{v^{2}}{gL}$. Level curves of the Froude number run parallel to the boundary between kinetic and quasi-static regions, for example, between regions I and II in Fig. 2A. As another example, the Reynolds number is the ratio between inertial force and viscous force in a flowing fluid, $Re=\frac{\rho vL}{\mu }$. Level curves of the Reynolds number run parallel to the boundary between kinetic and viscous regions, for example, between regions I and III in Fig. 2A. A third example is within the quasi-static region (region II), in which the ratio between gravitational and elastic forces (quantified by “specific modulus”) is the important dimensionless number. The phase shift between force and position thus illustrates which dimensionless quantity is most important for a given motion—showing, for example, that when the phase shift is 180°, Froude number is very relevant for a behavior (such as horse locomotion), whereas when the phase shift is close to 0, Froude number is not very relevant for a behavior (such as stick insect locomotion). Furthermore, because *ϕ* varies continuously over the entire space of limb lengths and cycle periods, it may facilitate meaningful comparison between apparently similar motions in which different forces dominate.

What needs to be measured experimentally to predict neural control patterns? Measuring *ϕ* between force and displacement while moving the limb in a cyclic pattern with frequency *ω* would enable an experimentalist to quantify the dominant force within a limb motion. Because this framework does not directly rely on muscle contraction properties, the limb can be moved by any sort of force in such an experiment, including muscle force, inertia, a mechanical manipulator, or an applied magnetic field (50, 51). Direct measurement of *ϕ* is important because although this study employed allometric scaling to predict *ϕ* at different scales, allometric scaling is approximate and does not explain all variability in mechanical parameters between species (though the overall predictions of the model are robust to variations in key parameter values; Fig. S6). Moreover, correlations between different forces and kinematics can be used to indirectly infer the distribution of muscle work into elastic potential, gravitational potential, kinetic, and viscous (dissipated) energy (12). To create a more detailed model of a limb segment, an experimentalist may approximate the model parameters $k_{r,\mathrm{elas}},\;k_{r,\mathrm{grav}}$, and $c_{r}$ by measuring the limb's mass, its length, and *ϕ* in response to two different forcing periods, *T*.

Analysis of phase shift also informs the construction of more accurate neurorobotic models of animals. Because our analysis does not consider muscle dynamics, robotic motions can also be classified by *ϕ*. Typical robot construction methods, in which massive segments are actuated by electric gearmotors, produce robots dominated by inertial and gravitational forces, much like large animals. Such a robot would likely exhibit values of *ϕ* near 0° during slow motions and 180° during rapid motions, which would be a poor model of a small arthropod, whose *ϕ* value should be between 0° and 90° in all contexts (Fig. 5A and B). As pointed out by Hooper (11), to make meaningful comparisons between an animal and a robotic model, it is important for the robot to match the fundamental relative physics of its inspiration (relative inertial, elastic, and viscous forces). In the future, engineers may construct more accurate and useful neurorobotic models of insects by altering the robot's mechanics (e.g. by adding springs that resist motor output (52)) and slowing its speed of operation to ensure that its *ϕ* values match the model animal's. Such alterations would guarantee the same energy allocation (although at different magnitude) between robot and animal. Matching energy allocation will both improve robots as models for animals and allow animal neural control patterns to be used more effectively in robots.

## Supplementary Material

pgad298_Supplementary_Data

## Data Availability

The data set and models used for this work will be made available online. The data can also be taken from the individual references cited. Simulations and parameters for this work can be found on this GitHub site: https://github.com/nicksz12/dynamicScaling.
