# Supplementary material for: Phase shift between joint rotation and actuation reflects dominant forces and predicts muscle activation patterns
Source: PNAS Nexus. 2023 Oct 10;2(10):pgad298. doi: 10.1093/pnasnexus/pgad298 (PMC10563792; doi:10.1093/pnasnexus/pgad298)
Supplement: pgad298_Supplementary_Data [file pgad298_supplementary_data.docx]

# Materials and Methods

## Model Setup and Assumptions

The model represents simplified geometry and dynamics of a mobile limb, e.g., the leg of a horse rotating about the shoulder or the leg of an insect rotating about its thorax-coxa joint. The dynamics of this system differ depending on whether the leg is in swing phase, during which the leg is moved anteriorly and does not support the body (protraction, Figure S1A), or in stance phase, during which the leg is moved posteriorly while supporting and propelling the body (retraction, Figure S1B). In both cases, the limb is assumed to have length $L$ with mass $m_{L}$ and moment of inertia $J_{L}$. The limb is assumed to operate in a gravitational field with acceleration $g$, and the rotation of the limb relative to the direction of gravity is measured by $\theta$. The limb is moved by an antagonistic pair of actuators, e.g., shoulder retractors and protractors, which together have total inherent elastic stiffness $k_{elas}$ and viscous damping $c$. As each actuator activates, it may deliver the force $F_{actuator}$ to the limb, which does work $W_{actuator}$ as the limb rotates. The subsequent results and analysis are concerned with where the actuator work *goes* (i.e., into the limb’s kinetic energy, into its potential energy, or dissipated as heat), not from whence the actuator work *comes* (e.g., from muscle, an external force during an experiment (Kathman and Fox 2019), or a robot actuator (Caldwell *et al.* 1993)) [Please note that all references cited in the Supplemental Materials are at the end of the supplement, and are cited in (Author, Date) format, unlike the references cited in the main manuscript].

The model differs between swing and stance. In swing, the leg is pinned at point $O$ and the body is considered “grounded”, that is, it does not move. Gravity and elasticity stabilize the leg, trying to return it to $\theta=0$. In stance, the leg is pinned to the body at point $O$ and to the ground at point $P$, and the body translates over the leg. In stance, elasticity stabilizes the leg, but gravity destabilizes the leg (note direction of $k_{L,grav}$ in Figure S1A and B). Furthermore, the body’s mass $m_{B}$ increases the magnitude of inertial and gravitational forces relative to those during the swing phase.

This analysis quantifies the relative size of three terms: a limb’s 1) kinetic energy, 2) elastic energy, and 3) viscous (dissipated) energy as a function of limb length and cycle time. Thus, our focus is the question “When an actuator adds energy to the system, how is that energy partitioned between these three quantities?”. The balance of these three terms, however, is not affected by the specific properties of the actuator providing that energy; rather, it is only a function of how the total energy is partitioned, which in turn depends on the passive properties of the limb (and body) and the cycle period of the motion. For example, in the viscous-dominated region (region III in Figure 2 in the manuscript), almost all actuator energy is viscously damped out by internal joint friction – regardless of whether the driving force is provided by muscle, motor, or any other actuator (for example, a magnet driving a haltere glued to an iron filing (Kathman and Fox 2019)). The viscous damping is a function of limb properties and is independent of actuator type. Moreover, the boundary between the viscous-dominated and quasi-static regions (region III and region II) is solely determined by cycle period and is also independent of actuator type. In general, independence of actuator type is true for all the regions shown in Figures 2-5 in the manuscript.

Actuator limitations certainly do affect the maximum limb speeds that are achievable by animals of certain sizes (see (Zajac 1989, Alexander 2005, 1995, Hooper 2012, Sutton *et al* 2019, Usherwood and Gladman 2020) for details). For example, while a 1 m long limb moving with a frequency of 100 Hz would be in the inertially dominated region I, muscle cannot provide enough power to move that much mass at that high a speed (Zajac, 1989; Ellington 1985). Thus, active muscle properties (e.g., force-velocity relationships) can restrict the achievable spaces shown in Figures 2-5 in the manuscript. Within each of the regions, however, active muscle properties do not affect how energy is distributed. Since the current study is focused only on energy partitioning and its effect on movement stability and phase-angle relationships, both of which are unaffected by actuator properties, consideration of detailed muscle properties is only addressed briefly in the Discussion of the manuscript as a basis for future work.

The dynamics of any linear spring-mass-damper system with one degree of freedom can be written in a familiar form in terms of the linear displacement of the actuator $x$. The actuator force, $F_{actuator}$, is equal to the sum of the passive dynamics: mass times acceleration, $m\ddot{x}$; damping coefficient times velocity, $c\dot{x}$; and elastic stiffness times displacement, $k_{elas}x$. These terms make up the equation of motion:

$F_{actuator}=F_{inertial}+F_{viscous}+F_{elastic}=m\cdot\ddot{x}+c\cdot\dot{x}+k_{elas}\cdot x$. (1)

To better model rotation around rotary joints, the equation is converted into rotary form by summing the moments of the forces about the joint, $O$ (see descriptions below). To facilitate this conversion, free body diagrams are presented in Figure S1C and D. Summing moments about $O$ changes the equation of motion in three ways. First, the equation is now in terms of $\theta$, the angle of the limb relative to the direction of gravity. Second, the constant parameters are converted into their rotary analogues (Figure S1A and B). Third, the rotary stiffness of the leg due to gravity, $k_{L,grav}$, is added. This term represents the limb’s tendency to hang in parallel with the direction of gravity and oscillate like a pendulum when perturbed. The moment applied by gravity is proportional to the limb rotation $\theta$ with an error less than or equal to 5% as long as $\left| \theta\right|\leq{30}^{\circ}$ (i.e. “small angle approximation” see justification below and Figure S2A), which is observed in the hip angles of many animals during locomotion (elephant: (Ren *et al* 2008); horse: (Roepstorff *et al* 2009); human: (Kadaba *et al* 1990); small mammals: (Fischer *et al* 2002); stick insects (Cruse and Bartling 1995); discoid cockroaches: (Bender *et al* 2010)). Because elastic moments and gravitational moments are both proportional to the joint rotation $\theta$, these two terms can be grouped together:

$M_{actuator}=J_{L}\cdot\ddot{\theta}+c_{r}\cdot\dot{\theta}+\left( k_{r,elas}+k_{L,grav} \right)\cdot\theta$. (2)

In stance, Eq. (2) must change to include the mass of the body in both the inertia and $k_{grav}$ terms. Furthermore, because gravity destabilizes posture in the stance phase, the sign of $k_{L,grav}$ must also change:

$M_{actuator}=\left( J_{B}+J_{L} \right)\cdot\ddot{\theta}+c_{r}\cdot\dot{\theta}+\left( k_{r,elas}-\left( k_{L,grav}+k_{B,grav} \right) \right)\cdot\theta$. (3)

Finally, because energy is a critical currency underlying animal behaviour, the rotary equation of motion is converted into a work-energy equation by integrating the equation of motion with respect to $\theta$. Again, each term in the work-energy equation is analogous to a term in the equation of motion (Equation 2): Actuator moment becomes actuator work; inertial moment becomes kinetic energy; viscous moment becomes dissipated energy; elastic and gravitational moments become elastic and gravitational potential energy. The amounts of energy (kinetic, and viscous) are plotted in the work-loop diagrams in Figure 2. When the leg is in swing, this is mathematically formulated as:

$W_{actuator}=\frac{1}{2}J_{L}\cdot\dot{\theta}^{2}+c_{r}\cdot\int\dot{\theta}^{2}dt+\frac{1}{2}\left( k_{r,elas}+k_{L,grav} \right)\cdot\theta^{2}$. (4)

When the leg is in stance, this is mathematically formulated as:

$W_{actuator}=\frac{1}{2}\left( J_{B}+J_{L} \right)\cdot\dot{\theta}^{2}+c_{r}\cdot\int\dot{\theta}^{2}dt+\frac{1}{2}\left( k_{r,elas}-\left( k_{L,grav}+k_{B,grav} \right) \right)\cdot\theta^{2}$. (5)

To simplify the parameterization of the model, we sought to express all masses, damping coefficients, and stiffnesses in terms of the limb length, $L$. Both empirical and theoretical evidence support that an animal’s mass scales nearly according to its volume, such that $m=m_{0}L^{3}$. The constant $m_{0}$ has units $\frac{kg}{m^{3}}$, and we approximated $m_{0}=12$ from measurements in the literature (Hemmingsen 1960). Because the moment of inertia of a slender rigid body rotating about its end is $J=\frac{1}{3}mL^{2}$, we approximate the moment of inertia of a leg as $J_{L}=\frac{1}{3}m_{0}L^{5}$. In our simplified model of stance, the body does not rotate, meaning that it functions as a point mass whose moment of inertia about the foot is $J_{B}=\frac{1}{2}m_{B}L^{2}$. The mass of the body is assumed to be a multiple of the mass of the leg, $m_{B}=a\cdot m_{L}$, where $5\leq a\leq20$ (Bennet Clark 1975, Crook *et al* 2008, Szczecinski *et al* 2018).

The gravitational stiffness of the limb is the ratio between the moment applied by gravity, $M_{grav}=\frac{1}{2}mgL\sin\theta,$ and the angle of the limb relative to the direction of gravity, $\theta$. However, because for most animals, the hip angle $\left| \theta\right|\leq{30}^{\circ}$ (Ren *et al.* 2008, Roepstorff *et al.* 2009, Kadaba *et al.* 1990, Fischer *et al.* 2002, Cruse and Bartling 1995, Bender *et al.* 2010) and mass is distributed approximately uniformly along the length of each leg segment, the moment applied to the leg by gravity is well-approximated by $M_{grav}=\frac{1}{2}mgL\theta$, meaning that $k_{L,grav}=\frac{M_{grav}}{\theta}=\frac{1}{2}mgL$. Substituting in the scaling relationship between mass and length, $k_{L,grav}=\frac{1}{2}m_{0}gL^{4}$. Because the body was treated as a point mass at approximately distance $L$ from the foot, $k_{B,grav}=m_{B}gL$.

The stiffness of an elastic material is proportional to its length, such that $k_{elas}=k_{0}L$, where $k_{0}$ is an empirically determined constant (Gere and Goodno 2001). This assumption of our model conflicts with the assumptions of some previous studies (Hooper *et al.* 2009) but is consistent with others (Garcia *et al* 2000). However, our assumption that $k_{elas}=k_{0}L$ is supported by two independent lines of reasoning: One from first principles of the mechanics of materials (Gere and Goodno 2001) and one from empirical measurements of muscle force (Zajac 1989).

First, from mechanics of materials, the tensile force $F$ with which a body (e.g., a muscle) resists change in length $\Delta L$ is equal to

$F=\frac{EA}{L}\cdot\Delta L$, (6)

where $E$ is the Young’s Modulus of the body’s constituent material, $A$ is the cross-sectional area of the body normal to the direction of elongation, and $L$ is the equilibrium length of the body (Gere and Goodno 2001). Note that this equation is in the form of Hooke’s Law: tensile force is proportional to elongation multiplied by the spring rate,

$k=\frac{EA}{L}$. (7)

Because $E$ is a bulk property of the body’s material, it is independent of a body’s geometry and does not scale with length. In contrast, scaling the body isometrically (McMahon 1973, Hemmingsen 1960, Alexander *et al* 1979) would cause $A$ to scale with $L^{2}$. Let us define a constant parameter $A_{0}$ that relates the cross-sectional area of a muscle to its length,

$A=A_{0}L^{2}$. (8).

Substituting Eq. 8 into Eq. 7, we obtain

$k=EA_{0}L$, (9)

which implies that the stiffness $k$ of any elastic body scales with its equilibrium length $L$. From Eq. (9), we obtain the scaling constant from the beginning of this paragraph,

$k_{0}=EA_{0}$. (10)

The second line of reasoning that supports the model’s assumption that muscle stiffness scales with $L$ is based on empirical measurements of the maximum muscle force an animal can exert relative to its body weight (Alexander 1985). The maximum force that an animal can exert, $F_{\max}$, normalized to that animal’s weight, $mg$, scales according to $m^{-\frac{1}{3}}$ over 9 orders of magnitude of mass (Alexander 1985). Here, we show that this can only be true if $k$ scales with $L$, and cannot be true if $k$ scales according to $L^{2}$. For an animal to exert its maximum force “involve[s] near-maximal stresses in the muscles concerned” (Alexander 1985), implying that the muscles are stretched to their maximal length (Zajac 1989).

Let us first assume that muscle stiffness scales with L and verify that this assumption matches Alexander 1985. Let us define maximum muscle strain $\epsilon_{\max}$, such that when a muscle is stretched to its maximum length, the force it exerts is

$F_{\max}=k_{0}L\cdot\Delta L=k_{0}L\cdot\left[ \left( 1+\epsilon_{\max} \right)-1 \right]\cdot L$. (11)

Recalling that $m=m_{0}L^{3}$ (McMahon 1973, Hemmingsen 1960), the normalized maximum force from (Alexander 1985) is calculated to be

$\frac{F_{\max}}{mg}=\frac{k_{0}\epsilon_{\max}L^{2}}{m_{0}L^{3}g}=\frac{k_{0}\epsilon_{\max}}{m_{0}gL}$. (12)

Finally, because we wish to see how Fmax scales against m, the mass scaling equation is rearranged to $L=\sqrt[3]{m/{m_{0}}}$ and substituted into Eq. (12) to reveal that if $k$ is assumed to scale with $L$,

$\frac{F_{\max}}{mg}=\frac{k_{0}\epsilon_{\max}}{m_{0}^{2/3}g}\cdot m^{-\frac{1}{3}}$, (13)

as reported by (Alexander 1985) and verifying the initial assumption that muscle stiffness scales with L.

Furthermore, the scaling of maximum muscle force reported by (Alexander 1985) cannot be produced if muscle stiffness $k$ is assumed to scale with $L^{2}$. Repeating the above analysis, one would calculate that

$F_{\max}=k_{0}L^{2}\cdot\Delta L=k_{0}L^{2}\epsilon_{\max}\cdot L$. (14)

Again recalling that $m=m_{0}L^{3}$ (McMahon 1973, Hemmingsen 1960), the normalized maximum force from (Alexander 1985) would be calculated as

$\frac{F_{\max}}{mg}=\frac{k_{0}\epsilon_{\max}L^{3}}{m_{0}L^{3}g}=\frac{k_{0}\epsilon_{\max}}{m_{0}g}$, (15)

suggesting no length-dependent scaling in the maximum force an animal can exert relative to its body weight. Because this directly contradicts the measurements reported in (Alexander 1985), we reject the assumption that muscle stiffness scales with $L^{2}$.

The effective rotary stiffness of the joint $k_{r,elas}$ is calculated from the linear stiffness of the muscle $k_{elas}$. Extending the limb segment in Figure S1A stretches the spring. The potential energy of the spring is $V_{elas}=\frac{1}{2}kx^{2}$. However, the potential energy of the spring needs to be expressed in terms of the rotary coordinate $\theta$, $V_{elas}=\frac{1}{2}k_{r,elas}\theta^{2}$, where $k_{r,elas}$ is unknown. If $\left| \theta\right|<0.5$ radian (${\approx30}^{\circ})$, then $x\approx r\theta$, with the maximum error in $V_{elas}$ being less than 10% (Figure S2B), which averages to only 3% over the entire stepping cycle (Figure S2C). Expressing $r$ as a fraction $b$ of $L$ yields the approximation that $x=bL\theta$. Substituting this expression into $V_{elas}$ enables one to solve for $k_{r,elas}$,

$V_{elas}=\frac{1}{2}k\left( bL\theta\right)^{2}=\frac{1}{2}kb^{2}L^{2}\theta^{2}=\frac{1}{2}k_{r,elas}\theta^{2}$. (16)

Substituting in the scaling relationship for $k,$ we obtain $k_{r,elas}=k_{0}b^{2}L^{3}$, where $b=r/L$. Empirically, ${10}^{-2}\leq b\leq{10}^{-1}$ (Williams *et al* 2008, Greene 1955, Guschlbauer *et al* 2007, Full and Ahn 1995). For our study, we set $b={10}^{-1.5}=0.032$, the geometric mean of the empirical range of $b$.

We used measurements reported in the literature to approximate the value of the constant $k_{0}$. In our model, $k_{r,grav}=\frac{1}{2}m_{0}gL^{4}$ and $k_{r,elas}=k_{0}b^{2}L^{3}$, meaning that when $L$ is large, gravitational forces drive the leg to its equilibrium orientation, but when $L$ is small, elastic forces drive the leg to its equilibrium angle. Figure S3 plots the relative size of gravitational and elastic stiffness as a function of $L$. By comparing the balance between $k_{r,grav}$ and $k_{r,elas}$ in the femur-tibia joint of a human and a stick insect, we can estimate an appropriate value for $k_{0}$. When a person hangs their tibia off a table and relaxes their muscles, 75% of the torque driving the tibia to hang vertically is produced by gravity and 25% of the torque is produced by the elastic properties of the muscles in the thigh (Stein *et al* 1996). In contrast, the femur-tibia joint of a stick insect is so stiff that the tibia will not hang with gravity, even when the body is inverted and the muscles in the femur are not actively contracting (Hooper *et al* 2009). This suggests that in the stick insect nearly 100% of the torque acting on the tibia is produced by the elastic properties of the muscles in the femur. Setting $k_{0}=12\times{10}^{3}\frac{N}{m^{2}}$ achieves the correct balance between gravitational and elastic stiffness as observed in animal leg joints (Stein *et al* 1996, Hooper *et al* 2009).

Another consequence of $k_{r,grav}$ and $k_{r,elas}$ scaling differently is that at different length scales, the undamped natural frequency, and thus the most energetically efficient movement speed, scales with a different exponent of $L$. For a leg in swing, the undamped natural frequency $\omega_{n}=\sqrt{\frac{k_{r,grav}+k_{r,elas}}{J_{L}}}$. One might expect the duration of a leg’s swing to reflect the undamped natural period of its motion (i.e., one half a pendulum swing), $T_{n}=\frac{2\pi}{\omega_{n}}$. For a large leg, $k_{r,grav}\gg k_{r,elas}$, so $T_{n}\approx\sqrt{\frac{\frac{1}{3}\cdot m_{0}\cdot L^{5}}{\frac{1}{2}\cdot m_{0}g\cdot L^{4}}}=\sqrt{\frac{2}{3g}}\cdot\sqrt{L}$. However, for a small leg, $k_{r,grav}\ll k_{r,elas}$, so $T_{n}\approx\sqrt{\frac{\frac{1}{3}\cdot m_{0}\cdot L^{5}}{k_{0}{b^{2}L}^{3}}}=\sqrt{\frac{\frac{1}{3}\cdot m_{0}}{k_{0}b^{2}}}\cdot L$. Figure S4 plots $T_{n}$ *versus* $L$ to illustrate this scale-dependent change in scaling exponent. This effect may explain why previous studies have reported equally good least-squares fits between $L$ and swing phase duration, whether $T_{n}$ was assumed to scale with $\sqrt{L}$ or $L$ (Figure 8A of (Hooper *et al* 2009)).

We found no comprehensive review of viscous damping coefficients in animal leg joints across length scales in the literature. Thus, we applied first principles to seek an appropriate scaling relationship for viscous damping, then used measurements from the literature to approximate undetermined coefficients. We recognize that moving through a fluid medium will produce forces that resist motion. However, for legged locomotion in air, these forces are expected to be inconsequentially small compared to inertia, gravity, and elasticity (Hooper 2012, Alexander 1976). Thus, we are only concerned with intrinsic damping arising from within the body. The viscous force acting on a solid body, e.g., the viscous drag on a muscle as it contracts through the surrounding wet fascia, is

$F_{viscous}=\frac{1}{2}\cdot\rho_{fluid}\cdot\dot{x}^{2}\cdot C_{d}\cdot L^{2}$, (17)

where $\rho_{fluid}$ is the density of the fluid,  $\dot{x}$ is the relative speed of the body through the fluid, $C_{d}$ is the empirically determined coefficient of drag, and $L$ is the characteristic length of the system (Turns 2006). We assume that the fluid has the properties of water. Let us introduce the Reynold’s number, $Re$, a dimensionless ratio between the inertia and viscosity of a fluid,

$Re=\frac{\rho_{fluid}\cdot\dot{x}\cdot L}{\mu_{fluid}}$, (18)

where $\mu_{fluid}$ is the dynamic viscosity of the fluid. Flow is laminar if $Re$ is small and flow is turbulent if $Re$ is large. Because we assume joint damping is due to wet muscle and fascia sliding past each other in close contact (i.e., small $L$), we further assume that $Re$ is small. Empirical studies show that when $Re$ is sufficiently small, $C_{d}$ in Equation 17 is inversely proportional to $Re$ (Clancy 1975),

$C_{d}=A\cdot\left( Re \right)^{-1}$, (19)

where $A$is a constant. Substituting Equations 18 and 19 into Equation 17 and simplifying, we find that

$F_{viscous}=c_{0}\cdot L\cdot\dot{x}$, (20)

where $c_{0}=\frac{1}{2}\cdot\mu_{fluid}\cdot A$. Thus, Equation 20 suggests that the viscous force experienced by a muscle being dragged through a wet enveloping material should scale with the length scale of the system given our assumptions. Empirical data must be used to determine $c_{0}$, but since studies in the literature report the damping parameter of the joint’s rotation, we must first develop an expression for the rotary damping of the joint.

The effective damping coefficient of the joint $c_{r}$ is calculated from the linear damping of the muscle $c$. This process mirrors that applied to calculate $k_{r,elas}$ and yields a parallel result,

$c_{r}=c_{0}b^{2}L^{3}$, (21)

suggesting that the rotary viscous damping coefficient of a joint should scale with the length scale cubed. To validate this relationship, we used published data regarding joint damping in the human hip, human knee (Stein *et al* 1996), human ankle (Weiss *et al* 1988), human finger (Hajian and Howe 1997), locust femur-tibia joint (Zakotnik *et al* 2006), and cockroach femur-tibia joint (Garcia *et al* 2000) and performed a least-squares fit to extract parameter $c_{0}$ (Figure 1H). We calculated the value $c_{0}=1.31\times{10}^{3}\frac{Ns}{m^{2}}$ for this study. All parameter values in the model are listed in Table S1.

## Calculation and Scaling of the Phase Shift, $\phi$

To predict whether actuator work is predominantly converted into the limb’s kinetic energy, dissipated due to viscous effects, or converted into the limb’s potential energy, we calculated the phase shift between the actuator’s force and the limb’s angle. The phase shift is meaningful because each force under consideration acts at a different phase relative to the limb displacement. The moments that result from increasing the potential energy of the limb (i.e., elastic and gravitational) are proportional to limb angle, and thus have a phase shift of $0^{\circ}$ relative to limb angle. If the limb oscillates such that $\theta\left( t \right)=sin(\omega t)$, then these moments also vary $M_{elas+grav}=k_{elas+grav}\cdot sin(\omega t)$. In contrast, the moments that dissipate energy through viscosity are proportional to the limb’s angular velocity,  $\dot{\theta}\left( t \right)=\omega\cdot cos(\omega t)$. Note that cosine is a phase-shifted sine wave, i.e., $\cos\left( \omega t \right)=sin(\omega t+{90}^{\circ})$, meaning that the phase shift of viscous moments is ${90}^{\circ}$ relative to limb angle. Extending this logic, the moments that result from increasing the kinetic energy of the limb (i.e., inertial) are proportional to the limb’s angular acceleration, resulting in a phase shift of ${180}^{\circ}$ relative to limb displacement. Thus, by weighting the phase shift of each force by its relative magnitude (i.e., a vector sum), the resultant phase shift between the actuator moment and limb angle can be calculated analytically (Rao 2011). In swing, the phase shift is

$$\phi_{sw}=arctan2 \left( c_{r}\omega, k_{elas}+k_{L,grav}-J_{L}\omega^{2} \right),$$

and in stance, the phase shift is

$$\phi_{st}=arctan2 \left( c_{r}\omega,k_{elas}-k_{L,grav}-k_{B,grav}-\left( J_{L}+J_{B} \right)\omega^{2} \right),$$

where arctan2(y,x) is the two-argument tangent function that has range $\left[ -\pi,\pi\right].$The phase shift explicitly depends on the frequency of the periodic motion. However, rather than expressing $\phi$ in terms of the limb oscillation frequency $\omega$, we find it more intuitive to think about a motion in terms of its cycle period, $T=\frac{2\pi}{\omega}$. Furthermore, we have established that all constant parameters depend on limb length, $L$. Thus, the phase shift $\phi$ quantifies the distribution of the limb’s and body’s energy, and $\phi$ can be expressed in terms of $T$, $L$, and empirically determined constants, enabling us to explain and predict differences in locomotion strategies across several orders of magnitude of movement cycle periods and limb lengths:

$\phi_{sw}=arctan2 \left( c_{0}b^{2}\cdot L^{3}\cdot\frac{2\pi}{T},k_{0}b^{2}L^{3}+\frac{1}{2}m_{0}gL^{4}-\frac{1}{3}m_{0}L^{5} \cdot\left( \frac{2\pi}{T} \right)^{2} \right)$,

$\phi_{st}=arctan2 \left( c_{0}b^{2}\cdot L^{3}\cdot\frac{2\pi}{T},k_{0}b^{2}L^{3}-\left( \frac{1}{2}+a \right)m_{0}gL^{4}-\left( \frac{1}{3}+a \right)m_{0}L^{5} \cdot\left( \frac{2\pi}{T} \right)^{2} \right)$,

where $a=\frac{m_{B}}{m_{L}}$.

To visually represent how energy is partitioned during walking depends on $T$ and $L$, the manuscript frequently plots $\phi$ *versus* $T$ and $L$ *via* contour plots. This is done for two reasons. First, a contour plot “flattens” the three-dimensional surface of $\phi$ to be amenable to illustration on a page. Second, contours of $\phi$ represent behaviours of varying $T$ and $L$ for which actuator work is partitioned into potential, viscous, and kinetic energy in the same way. Thus, behaviours that lie along the same contour are dynamically similar to one another. Note that although contour plots visualize the data as discrete level curves, $\phi$ varies continuously with $T$ and $L$ in both swing and stance (Figure S5). Furthermore, despite the fact that $\phi$ is continuous across the entire parameter space, three distinct plateaus exist in which relatively large (i.e., order of magnitude) changes in $T$ and $L$ cause imperceptible changes in $\phi$. The existence of these plateaus justifies our use of three primary regions: kinetic, viscous, and quasi-static.

How sensitive is $\phi$ to the parameters within the model? Figure S6 plots the contours of $\phi$ for varying values of the constant parameters in the model (listed with their values in Table S1). The parameters $m_{0}$, $c_{0}$, and $k_{0}$ were separately increased by an order of magnitude and decreased by an order of magnitude. Increasing a parameter increases the relative size of the associated region on the plot. For example, Figure S6A shows that increasing $m_{0}$ increases the area of the kinetic region (where $\phi$ approaches ${180}^{\circ}$, i.e., yellow contours) and Figure S6B shows that decreasing $m_{0}$ decreases the area of the kinetic region. Likewise, increasing $c_{0}$ increases the area of the viscous region (where $\phi$ approaches ${90}^{\circ}$, i.e., orange contours in Figure S6C) and decreasing $c_{0}$ decreases the area of this region (Figure S6D). Finally, increasing $k_{0}$ increases the area of the quasi-static region (where $\phi$ approaches $0^{\circ}$, i.e., red contours in Figure S6E) and decreasing $k_{0}$ decreases the area of this region (Figure S6F). However, despite these changes, the overall contour shapes do not change. In each case, the contours separating the kinetic and viscous regions have the same slope, the contours separating the viscous and quasi-static regions have the same (vertical) slope, and all contours converge as both $T$ and $L$ increase. No apparent bifurcation of the system takes place as these constants change, suggesting that while the parameter values in Table S1 may be refined, the features of this framework are robust to large changes in parameter values.

The sensitivity of $\phi$ to changes in $b$, the actuator’s nondimensional lever arm on the limb (i.e., fraction of the limb length at which the muscle attaches) was also evaluated (Figure S6G and H). The parameter $b$ was increased by half an order of magnitude and decreased by half an order of magnitude, because this is the physiological range for this value (${10}^{-2}\leq b\leq{10}^{-1}$ (Williams *et al* 2008, Greene 1955, Guschlbauer *et al* 2007, Full and Ahn 1995)). As seen for changes to the other parameter values, changing $b$ does not significantly alter the shape of the $\phi$ contours. In fact, because $b$ appears in the expressions for rotary joint stiffness (Eq. 16) and damping (Eq. 21) but not the moment of inertia, increasing $b$ has the same effect as decreasing $m_{0}$ (cf. Figure S6G and Figure S6B).

## Supplementary data for Figure 4

The following process generated the predicted EMGs in Figure 4 of the manuscript. The hip joint is assumed to progress with constant velocity from the beginning of stance to the end of stance, then return to the start angle following a cubic polynomial (Figure S7A). This profile resembles the motions reported in several animals, including human and stick insect (Nilsson *et al* 1985, Dallmann *et al* 2019). The kinematic profile was fed through the model, from which the elastic, gravitational, viscous, and inertial torques at the joint were calculated (Figure S7B-D). The sum of these torques represent the torque that the actuator must apply (“net torque” in Figure S7B-D). The torque curve was split into two separate curves: the positive values, and the rectified negative values. The positive values represent the torque applied by the joint extensor actuator and the rectified negative values represent the torque applied by the joint flexor actuator. These values were smoothed with a first-order low-pass filter with half-power frequency 50 Hz (Hooper *et al* 2009). Furthermore, because there is a delay of approximately 50 ms between EMG onset and muscle force produced (Roberts and Gabaldon 2008), each curve was advanced in time by 50 ms. Due to the fast stepping frequency of the fruit fly, the filter frequency and delay time were adjusted to 500 Hz and 8 ms (Azevedo *et al* 2020).

## Implications for the spring-loaded inverted pendulum (SLIP) model in this analysis.

We included a linear spring in the stance phase of our model to estimate how much our energetics curves would be affected if the limb was modeled as a spring-loaded inverted pendulum (SLIP; Full and Koditschek, 1999) instead of modeling it simply as an inverted pendulum. In both models, actuator work across the joint is distributed across the horizontal (which accelerates the center of mass, stores energy in the elastic tissues of the joint, and is dissipated by damping in the joint), and the vertical (which stores energy in the elastic tissues of the joint, increases gravitational potential energy, and is dissipated by damping in the joint). SLIP inherently describes the elastic and viscous properties of the knee and assumes the hip is passive. This is critical to bouncing gaits such as running. However, our analysis focuses on the hip, which is actuated during locomotion. Furthermore, we are concerned with how the parameters within the hip relate to the motion of the joint, i.e., rotation, whereas the SLIP model abstracts away the rotational motion of the knee joint.

# Figures


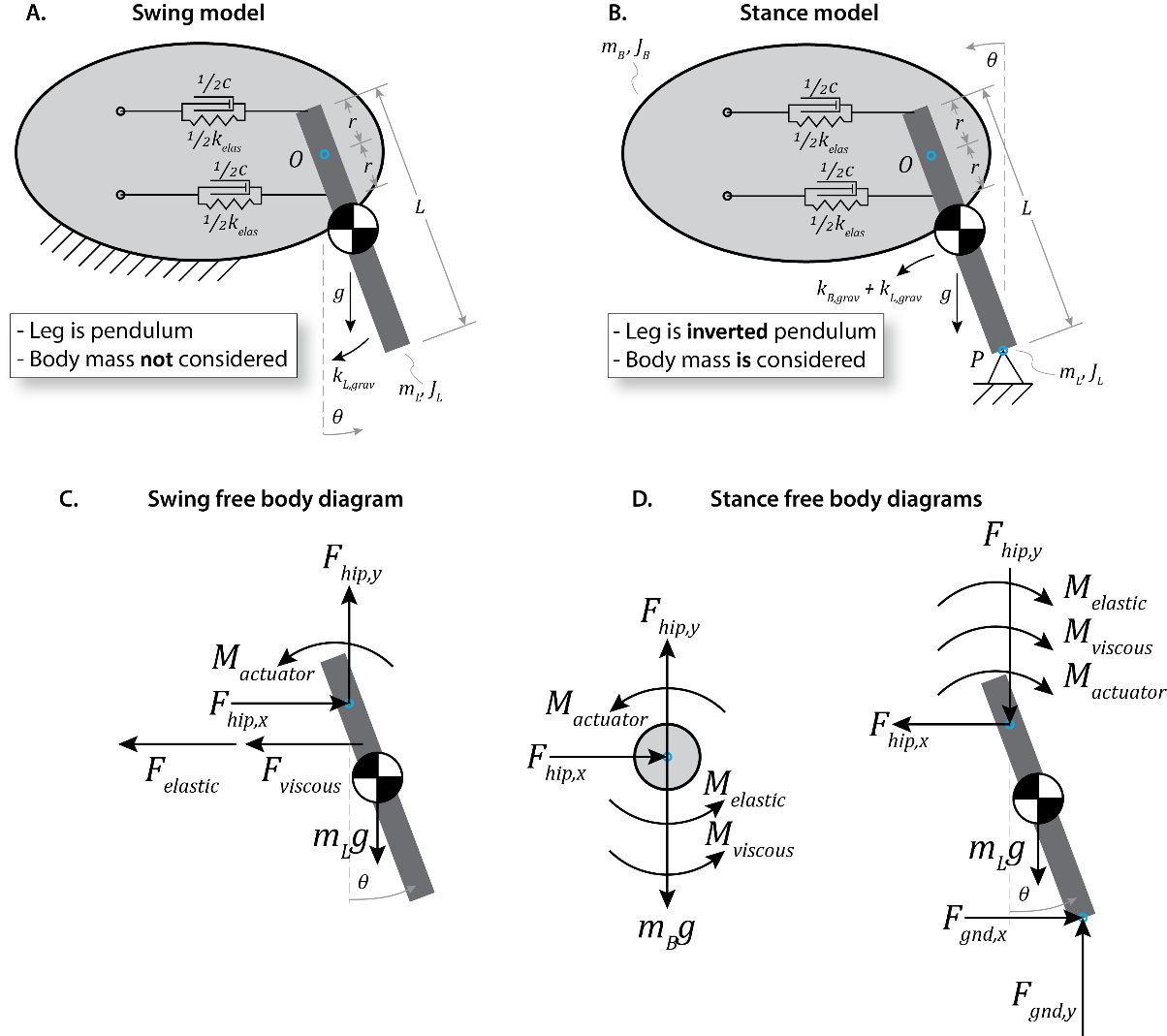


Figure S1 – Expressing the model components as free body diagrams. A. Diagram illustrating the key model measurements and components in swing. B. Diagram illustrating the key model measurements and components in stance. C. Free body diagram of the leg in swing. D. Free body diagrams of the body and leg in stance. Note that the body is treated as a point mass that cannot rotate but can translate forward.


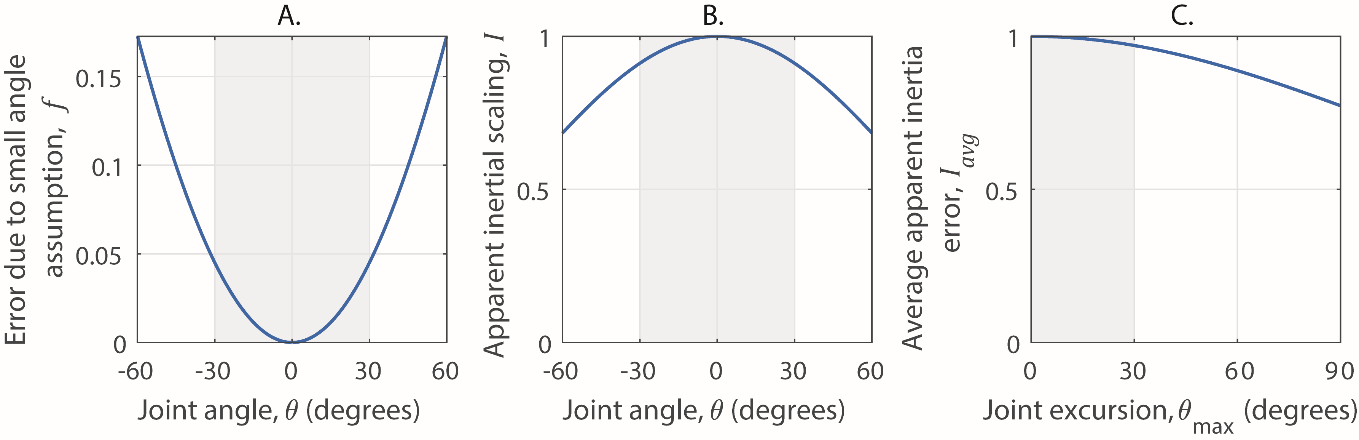


Figure S2 – Plots quantifying error introduced into our model by small angle approximation. A. Plot of the percent error $f$ between $\theta$ and $\sin\theta$, $f=\left( \frac{\theta-\sin\theta}{\theta} \right)$. The region of “small angles”, $\left| \theta\right|\leq{30}^{\circ}$, is shaded. B. Multiplicative impact on the model’s inertia of assuming $\sin\theta=\theta$. By approximating the rotary elastic potential energy as $V_{r,elas}=\frac{1}{2}k_{0}s^{2}\theta^{2}$ instead of $\frac{1}{2}k_{0}s^{2}\sin^{2} \theta$, and computing the kinetic energy exactly as $T=\frac{1}{2}J_{0}\dot{\theta}^{2}$, the model underestimates the inertia present in the system $I$at extreme angles, $I=\left( \frac{\sin\theta}{\theta} \right)^{2}$. C. The underestimation of inertia averaged over the entire range of motion, $I_{avg}=\frac{1}{2\theta}\cdot\int_{-\theta_{max}}^{\theta_{max}} \left( \frac{\sin\theta}{\theta} \right)^{2}\cdot dx$, reveals that the model approximates limb inertia to within 97% of its actual value for oscillations where $\left| \theta_{max} \right|\leq{30}^{\circ}$, and within 75% of its actual value for the largest amplitude oscillations possible.


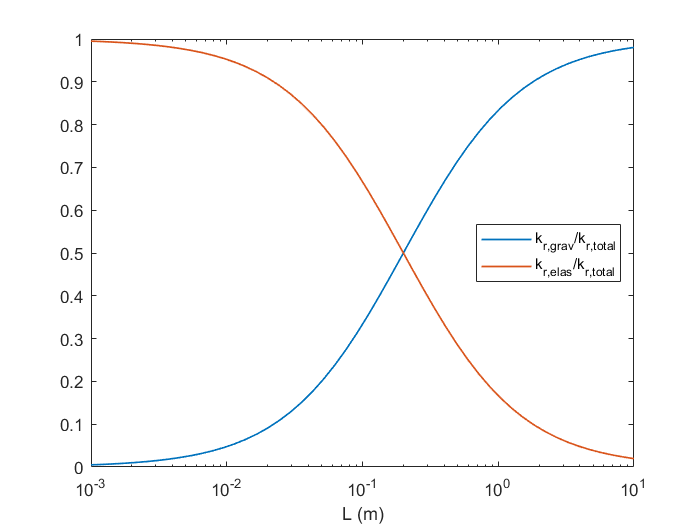


Figure S3 – Plot of the relative contribution of elastic (red) and gravitational (blue) potential energy to a system as a function of its length $L$. For small animals, most of the limb’s potential energy is stored in elastic elements; for large animals, most of the limb’s potential energy is stored in the posture of the leg.


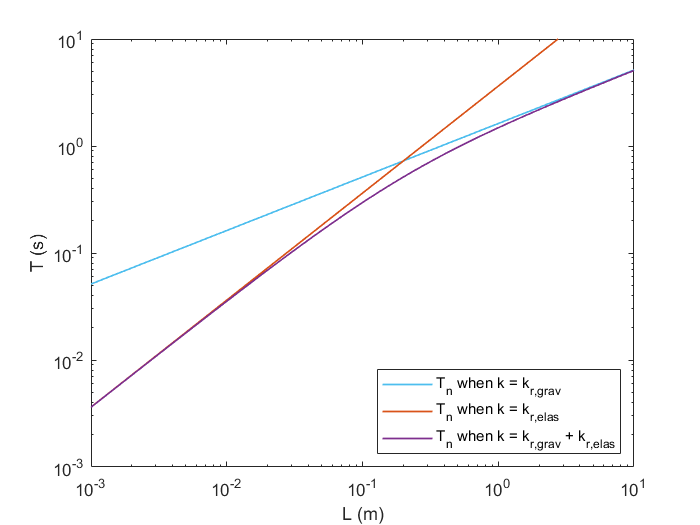


Figure S4 – The natural period $T_{n}$ does not scale uniformly with $L$. When potential energy is stored primarily as elastic potential energy ($L<{10}^{-1}$), $T_{n}\propto L$. In contrast, when potential energy is stored primarily as gravitational energy ($L>{10}^{0}$), $T_{n}\propto\sqrt{L}$.


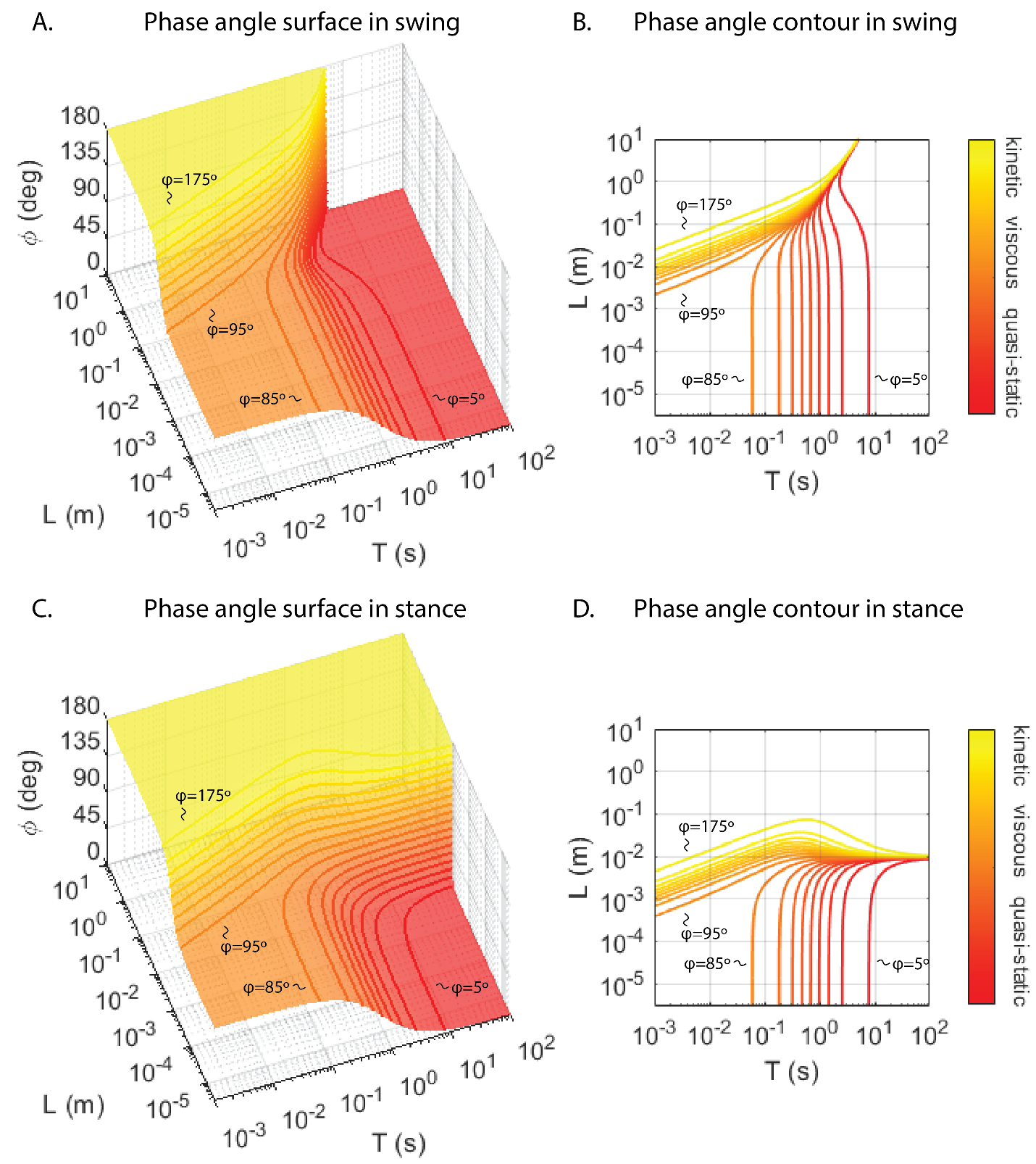


Figure S5 – Phase angle $\phi$ varies continuously with $T$ and $L$ in both stance and swing but exhibits plateaus where $\phi$ is insensitive to changes in $T$ and $L$. A. Surface plot of the phase angle during swing. The surface is colour-coded according to $\phi$. Contour lines are superimposed, spaced every ${10}^{\circ}$. B. Contour plot in which the plot from A is “flattened” into two dimensions. Despite the discrete contour lines, $\phi$ varies continuously. C. Surface plot of the phase angle during stance. The surface is colour-coded according to $\phi$. Contour lines are superimposed. D. Contour plot in which the plot from A is “flattened” into two dimensions. Despite the discrete contour lines, $\phi$ varies continuously.


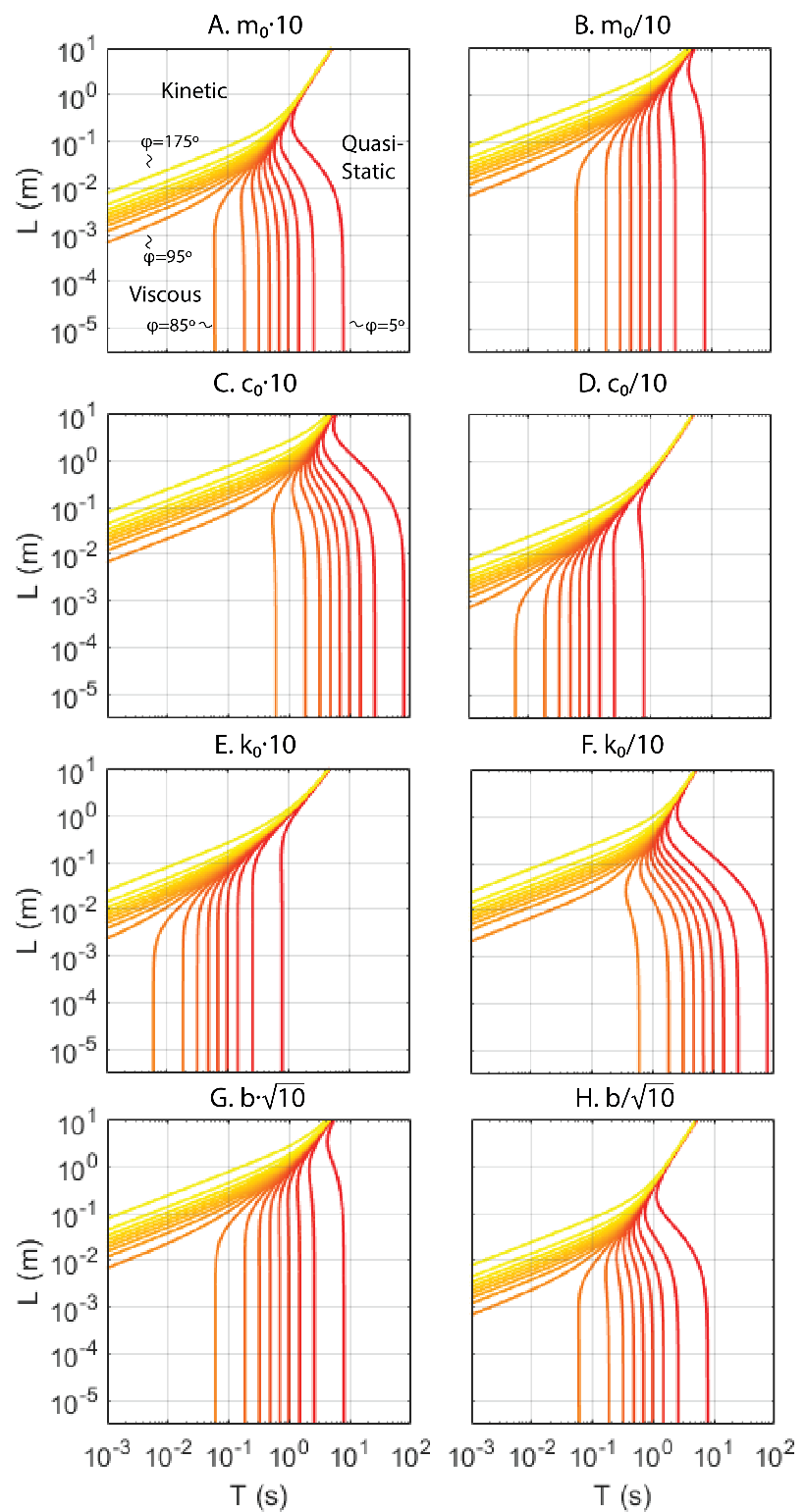


Figure S6 – Plots of the phase angle of a leg in swing from Figure S5B in which the parameters listed in Table S1 are varied. A. Contour lines indicating $\phi=5^{\circ}, {15}^{\circ},\ldots,{175}^{\circ}$. The quasi-static, viscous, and kinetic regions are indicated by the paucity of contour lines. Increasing the limb mass by an order of magnitude shifts the viscous-inertial boundary downward relative to Figure S5B but does not affect its slope. B. Decreasing the mass by an order of magnitude shifts the viscous-inertial boundary upward relative to Figure S5B but does not affect its slope. C. Increasing the damping by an order of magnitude shifts the viscous-inertial boundary upward and the viscous-quasi-static boundary rightward relative to Figure S5B but does not affect their slopes. D. Decreasing the damping by an order of magnitude shifts the viscous-inertial boundary downward and the viscous-quasi-static boundary leftward relative to Figure S5B but does not affect their slopes. E. Increasing the stiffness by an order of magnitude shifts the viscous-quasi-static boundary leftward rightward relative to Figure S5B but does not affect its slope. F. Decreasing the stiffness by an order of magnitude shifts the viscous-quasi-static boundary rightward relative to Figure S5B but does not affect its slope. G. Increasing the moment arm of the viscous and elastic elements by half an order of magnitude has the same effect as decreasing the mass by an order of magnitude. H. Decreasing the moment arm of the viscous and elastic elements by half an order of magnitude has the same effect as increasing the mass by an order of magnitude.


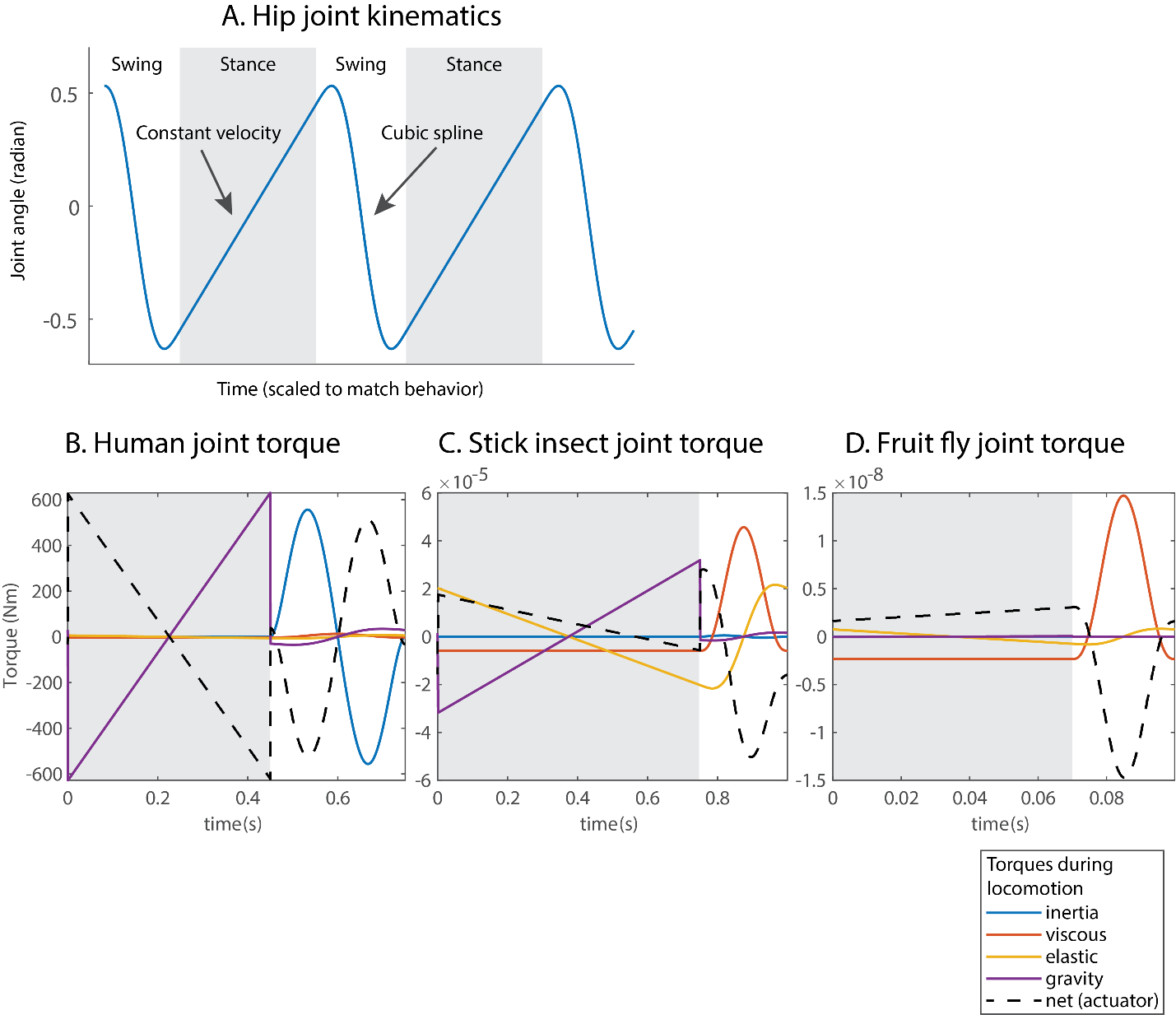


Figure S7 – Plots of the model’s joint angle and joint torques. A. The joint angle is assumed sweep 1 radian (approximately ${60}^{\circ}$) throughout stance at a constant velocity (determined by the walking speed), then return to the starting angle along a cubic spline to ensure the curve is continuous and smooth. B. In the human, the dominant torque in stance is gravity and the dominant torque in swing is inertia. The muscles must apply the net torque that counteracts these torques. C. In the stick insect, torques due to gravity, viscosity, and elasticity contribute to the net torque applied by the joint in stance. In swing, only viscosity and elasticity meaningfully contribute. D. In the fruit fly, torques due to viscosity dominate the entire motion. As a result, the net torque predicted for the fruit fly varies dramatically from the other animals investigated.

# Tables

Table S1 – Parameter values in the model.

| Parameter | Proportionality constant | Value | References |
| --- | --- | --- | --- |
| Mass | $m_{0}$ | $12\frac{kg}{m^{3}}$ | (McMahon 1973, Hemmingsen 1960) |
| Viscous damping | $c_{0}$ | $1.31\times{10}^{3}\frac{Ns}{m^{2}}$ | (Stein *et al* 1996, Weiss *et al* 1988, Hajian and Howe 1997, Zakotnik *et al* 2006, Garcia *et al* 2000) |
| Spring rate | $k_{0}$ | $12\times{10}^{3}\frac{N}{m^{2}}$ | (Stein *et al* 1996, Hooper *et al* 2009) |
| Lever arm | $b$ | ${10}^{-1.5}=0.032$ | (Williams *et al* 2008, Greene 1955, Guschlbauer *et al* 2007, Full and Ahn 1995) |

Table S2 – Table of walking animals and their period. Each species has a minimum and maximum period of motion, reported in seconds. The reference and the figure or table from which each value was drawn is listed for each value.

| Species | Min $\boldsymbol{T}$ (s) | Max $\boldsymbol{T}$ (s) | Reference | Location |
| --- | --- | --- | --- | --- |
| Horse | 880 x 10^-3^ | 1.34 | (Hildebrand 1959, Hooper *et al* 2009) | Body text |
| Human | 500 x 10^-3^ | 1 | (Grillner *et al* 1979) | Fig. 1 |
| Cat | 250 x 10^-3^ | 700 x 10^-3^ | (Grillner 1975) | Fig. 2 |
| Rat | 200 x 10^-3^ | 600 x 10^-3^ | (Hruska *et al* 1979) | Fig. 6 |
| Stick insect | 600 x 10^-3^ | 1.8 | (Cruse and Bartling 1995) | Tab. 1 |
| Mouse | 100 x 10^-3^ | 330 x 10^-3^ | (Herbin *et al* 2007) | Fig. 2 |
| American cockroach | 44 x 10^-3^ | 667 x 10^-3^ | (Delcomyn 1971) | Figs. 1, 4 |
| Fruit fly | 50 x 10^-3^ | 130 x 10^-3^ | (Wosnitza *et al* 2013) | Fig. 3 |

Table S3 – Definitions of symbols used in this manuscript.

| Symbol definitions |
| --- |
| $\boldsymbol{a}$, ratio between the mass of the body and mass of the leg, i.e., $\boldsymbol{a=}\boldsymbol{m}_{\boldsymbol{B}}\boldsymbol{/}\boldsymbol{m}_{\boldsymbol{L}}$ |
| $\boldsymbol{b}$, the nondimensional lever arm of the viscoelastic elements, i.e., $\boldsymbol{r/L}$. |
| $\boldsymbol{c}$, linear damping coefficient of viscous elements |
| $\boldsymbol{c}_{\boldsymbol{0}}$, viscous damping proportionality constant |
| $\boldsymbol{c}_{\boldsymbol{r}}$, rotary viscous damping coefficient of the joint |
| $\boldsymbol{E}$, Young’s modulus |
| $\boldsymbol{F}$, force applied by the actuator to the limb |
| $\boldsymbol{g}$, gravitational acceleration |
| $\boldsymbol{J}_{\boldsymbol{B}}$, moment of inertia of the body mass about the foot |
| $\boldsymbol{J}_{\boldsymbol{L}}$, moment of inertia of the leg about its pivot point (joint in swing, foot in stance) |
| $\boldsymbol{k}_{\boldsymbol{0}}$, elastic stiffness proportionality constant |
| $\boldsymbol{k}_{\boldsymbol{elas}}$, linear stiffness of elastic elements |
| $\boldsymbol{k}_{\boldsymbol{B,grav}}$, rotary gravitational stiffness of the joint due to the body’s mass |
| $\boldsymbol{k}_{\boldsymbol{L,grav}}$, rotary gravitational stiffness of the joint due to the leg’s mass |
| $\boldsymbol{k}_{\boldsymbol{r,elas}}$, rotary elastic stiffness of the joint |
| $\boldsymbol{L}$, the length of the limb |
| 𝑀, moment applied to the actuator by the limb. |
| $\boldsymbol{m}_{\boldsymbol{0}}$, mass proportionality constant |
| $\boldsymbol{m}_{\boldsymbol{B}}$, mass of the body |
| $\boldsymbol{m}_{\boldsymbol{L}}$, mass of the leg |
| $\boldsymbol{r}$, the lever arm of the viscoelastic elements |
| $\boldsymbol{T}$, the period of oscillation of the limb |
| $\boldsymbol{V}$, potential energy |
| $\boldsymbol{\theta}$, joint angle |
| $\dot{\boldsymbol{\theta}}$, joint angular velocity, i.e., $\boldsymbol{\omega}$ |
| $\ddot{\boldsymbol{\theta}}$, joint angular acceleration |
| $\boldsymbol{W}$, work |
| $\boldsymbol{\phi}$, phase shift between the limb’s angle $\boldsymbol{\phi}$ and the actuator moment $\boldsymbol{M}$ |
| $\boldsymbol{x}$, linear stretch of viscoelastic elements |

# References Cited in Supplement

Alexander R M 1995 Leg design and jumping technique for humans, other vertebrates and insects Philos. Trans. R. Soc. London. Ser. B Biol. Sci. 347 235–48 Online: https://royalsocietypublishing.org/doi/10.1098/rstb.1995.0024

Alexander R M 1976 Mechanics of bipedal locomotion Zoology (Elsevier) pp 493–504 Online: http://dx.doi.org/10.1016/B978-0-08-018767-9.50047-0

Alexander R M N 2005 Models and the scaling of energy costs for locomotion J. Exp. Biol. 208 1645–52

Alexander R M N 1985 Operation of the system for development of force, speed and power J. Exp. Biol. 115 231–8 Online: http://jeb.biologists.org/content/jexbio/115/1/231.full.pdf

Alexander R M N, Jayes A S, Maloiy G M O and Wathuta E M 1979 Allometry of the limb bones of mammals from shrews (Sorex) to elephant (Loxodonta) J. Zool. 189 305–14

Azevedo A W, Dickinson E S, Gurung P, Venkatasubramanian L, Mann R S and Tuthill J C 2020 A size principle for recruitment of drosophila leg motor neurons Elife 9 1–36

Bender J A, Simpson E M and Ritzmann R E 2010 Computer-assisted 3D kinematic analysis of all leg joints in walking insects. PLoS One 5

Bennet Clark H C 1975 The energetics of the jump of the locust Schistocerca gregaria J. Exp. Biol. 63 53–83

Caldwell D G, Razak A and Goodwin M 1993 Braided Pneumatic Muscle Actuators IFAC Proc. Vol. 26 522–7 Online: https://linkinghub.elsevier.com/retrieve/pii/S1474667017493542

Clancy L J 1975 Aerodynamics, John Wiley and Sons New York

Crook T C, Cruickshank S E, Mcgowan C M, Stubbs N, Wakeling J M, Wilson A M and Payne R C 2008 Comparative anatomy and muscle architecture of selected hind limb muscles in the Quarter Horse and Arab J. Anat. 212 144–52

Cruse H and Bartling C 1995 Movement of joint angles in the legs of a walking insect, Carausius morosus J. Insect Physiol. 41 761–71

Dallmann C J, Dürr V and Schmitz J 2019 Motor control of an insect leg during level and incline walking J. Exp. Biol. 222 jeb188748 Online: http://jeb.biologists.org/lookup/doi/10.1242/jeb.188748

Delcomyn F 1971 The Locomotion of the Cockroach Periplaneta Americana J. Exp. Biol. 54 443–52 Online: http://jeb.biologists.org/cgi/content/abstract/54/2/443

Ellington CP 1985 Power and efficiency of insect flight muscle. J. Exp. Biol. 115(1): 293-304

Fischer M S, Schilling N, Schmidt M, Haarhaus D and Witte H 2002 Basic limb kinematics of small therian mammals. J. Exp. Biol. 205 1315–38 Online: http://www.ncbi.nlm.nih.gov/pubmed/11948208

Full R and Ahn A 1995 Static forces and moments generated in the insect leg: comparison of a three-dimensional musculo-skeletal computer model with experimental measurements J. Exp. Biol. 198 1285–98 Online: http://www.ncbi.nlm.nih.gov/pubmed/9319155

Garcia M S, Kuo A D, Peattie A, Wang P and Full R J 2000 Damping And Size: Insights And Biological Inspiration Int. Symp. Adapt. Motion Anim. Mach. 1–7

Gere J M and Goodno B J 2001 Mechanics of Materials 5th Brooks Cole 780

Greene E C 1955 Anatomy of the Rat (New York, NY: Hafner Publishing Co.)

Grillner S 1975 Locomotion in vertebrates: central mechanisms and reflex interaction. Physiol. Rev. 55 247–304

Grillner S, Halbertsma J, Nilsson J and Thorstensson A 1979 The adaptation to speed in human locomotion Brain Res. 165 177–82

Guschlbauer C, Scharstein H and Buschges A 2007 The extensor tibiae muscle of the stick insect: biomechanical properties of an insect walking leg muscle J. Exp. Biol. 210 1092–108 Online: http://jeb.biologists.org/cgi/doi/10.1242/jeb.02729

Hajian A Z and Howe R D 1997 Identification of the Mechanical Impedance at the Human Finger Tip J. Biomech. Eng. 119 109–14 Online: https://asmedigitalcollection.asme.org/biomechanical/article/119/1/109/398500/Identification-of-the-Mechanical-Impedance-at-the

Hemmingsen A 1960 Energy metabolism as related to body size and respiratory surface, and its evolution. Reports Steno Meml. Hosp. 13 1–110

Herbin M, Hackert R, Gasc J-P and Renous S 2007 Gait parameters of treadmill versus overground locomotion in mouse Behav. Brain Res. 181 173–9 Online: https://linkinghub.elsevier.com/retrieve/pii/0024320579903898

Hildebrand M 1959 Motions of the running cheetah and horse. Journal of Mammalogy 40(4) 481-95. Online: https://www.doi.org/10.2307/1376265.

Hooper S L 2012 Body size and the neural control of movement Curr. Biol. 22 R318–22 Online: http://linkinghub.elsevier.com/retrieve/pii/S0960982212001996

Hooper S L, Guschlbauer C, Blümel M, Rosenbaum P, Gruhn M, Akay T and Büschges A 2009 Neural control of unloaded leg posture and of leg swing in stick insect, cockroach, and mouse differs from that in larger animals. J. Neurosci. 29 4109–19 Online: http://www.ncbi.nlm.nih.gov/pubmed/19339606

Hruska R E, Kennedy S and Silbergeld E K 1979 Quantitative aspects of normal locomotion in rats Life Sci. 25 171–9 Online: https://linkinghub.elsevier.com/retrieve/pii/0024320579903898

Kadaba M P, Ramakrishnan H K and Wootten M E 1990 Measurement of lower extremity kinematics during level walking J. Orthop. Res. 8 383–92 Online: http://link.springer.com/10.1007/978-1-4471-5451-8_100

Kathman N D and Fox J L 2019 Representation of haltere oscillations and integration with visual inputs in the fly central complex J. Neurosci. 39 4100–12

McMahon T 1973 Size and Shape in Biology: Elastic criteria impose limits on biological proportions, and consequently on metabolic rates Science (80-. ). 179 1201–4 Online: https://www.sciencemag.org/lookup/doi/10.1126/science.179.4079.1201

Nilsson J, Thorstensson A and Halbertsma J 1985 Changes in leg movements and muscle activity with speed of locomotion and mode of progression in humans Acta Physiol. Scand. 123 457–75

Nirody J A, Duran L A, Johnston D and Cohen D J 2021 Tardigrades exhibit robust interlimb coordination across walking speeds and terrains Proc. Natl. Acad. Sci. U. S. A. 118 1–9

Rao S S 2011 Mechanical Vibrations vol 33 (Prentice Hall)

Ren L, Butler M, Miller C, Paxton H, Schwerda D, Fischer M S and Hutchinson J R 2008 The movements of limb segments and joints during locomotion in African and Asian elephants J. Exp. Biol. 211 2735–51

Roberts T J and Gabaldon A M 2008 Interpreting muscle function from EMG: lessons learned from direct measurements of muscle force Integr. Comp. Biol. 48 312–20 Online: https://academic.oup.com/icb/article-lookup/doi/10.1093/icb/icn056

Roepstorff L, Egenvall A, Rhodin M, Byström A, Johnston C, van Weeren P R and Weishaupt M 2009 Kinetics and kinematics of the horse comparing left and right rising trot Equine Vet. J. 41 292–6

Stein R B, Zehr E P, Lebiedowska M K, Popovic D B, Scheiner A and Chizeck H J 1996 Estimating mechanical parameters of leg segments in individuals with and without physical disabilities IEEE Trans. Rehabil. Eng. 4 201–11

Sutton G P, Mendoza E, Azizi E, Longo S J, Olberding J P, Ilton M and Patek S N 2019 Why do large animals never actuate their jumps with latch-mediated springs? because they can jump higher without them Integr. Comp. Biol. 59 1609–18

Szczecinski N S, Bockemühl T, Chockley A S and Büschges A 2018 Static stability predicts the continuum of interleg coordination patterns in Drosophila J. Exp. Biol. jeb.189142 Online: http://jeb.biologists.org/lookup/doi/10.1242/jeb.189142

Turns S R 2006 Thermal-Fluid Sciences : an Integrated Approach (Cambridge University Press)

Usherwood J R and Gladman N W 2020 Why are the fastest runners of intermediate size? Contrasting scaling of mechanical demands and muscle supply of work and power: Scaling of demand, supply and top speeds Biol. Lett. 16 0–4

Weiss P L, Hunter I W and Kearney R E 1988 Human ankle joint stiffness over the full range of muscle activation levels J. Biomech. 21 539–44

Williams S B, Wilson A M, Rhodes L, Andrews J and Payne R C 2008 Functional anatomy and muscle moment arms of the pelvic limb of an elite sprinting athlete: The racing greyhound (Canis familiaris) J. Anat. 213 361–72

Wosnitza A, Bockemühl T, Dübbert M, Scholz H and Büschges A 2013 Inter-leg coordination in the control of walking speed in Drosophila J. Exp. Biol. 216 480–91 Online: http://jeb.biologists.org/cgi/doi/10.1242/jeb.078139

Zajac F E 1989 Muscle and tendon: properties, models, scaling, and application to biomechanics and motor control. Crit. Rev. Biomed. Eng. 17 359–411

Zakotnik J, Matheson T and Dürr V 2006 Co-Contraction and Passive Forces Facilitate Load Compensation of Aimed Limb Movements J. Neurosci. 26 4995–5007
